# Atomic Dispersed Co on NC@Cu Core‐Shells for Solar Seawater Splitting

**DOI:** 10.1002/adma.202406088

**Published:** 2024-10-14

**Authors:** Zhehao Sun, Shuwen Cheng, Xuechen Jing, Kaili Liu, Yi‐Lun Chen, Ary Anggara Wibowo, Hang Yin, Muhammad Usman, Daniel MacDonald, Soshan Cheong, Richard F. Webster, Lucy Gloag, Nicholas Cox, Richard D. Tilley, Zongyou Yin

**Affiliations:** ^1^ Research School of Chemistry The Australian National University Canberra Australian Capital Territory 2601 Australia; ^2^ School of Engineering The Australian National University Canberra Australian Capital Territory 2601 Australia; ^3^ Electron Microscope Unit Mark Wainwright Analytical Centre University of New South Wales Sydney New South Wales 2023 Australia

**Keywords:** Co single atom, local electric field, non‐noble metal photocatalyst, seawater splitting, solar to hydrogen

## Abstract

With freshwater resources becoming increasingly scarce, the photocatalytic seawater splitting for hydrogen production has garnered widespread attention. In this study, a novel photocatalyst consisting of a Cu core coated is introduced with N‐doped C and decorated with single Co atoms (Co‐NC@Cu) for solar to hydrogen production from seawater. This catalyst, without using noble metals or sacrificial agents, demonstrates superior hydrogen production effficiency of 9080 µmolg^−1^h^−1^, i.e., 4.78% solar‐to‐hydrogen conversion efficiency, and exceptional long‐term stability, operating over 340 h continuously. The superior performance is attributed to several key factors. First, the focus‐light induced photothermal effect enhances redox reaction capabilities, while the salt‐ions enabled charge polarization around catalyst surfaces extends charge carrier lifetime. Furthermore, the Co─NC@Cu exhibits excellent broad light absorption, promoting photoexcited charge production. Theoretical calculations reveal that Co─NC acts as the active site, showing low energy barriers for reduction reactions. Additionally, the formation of a strong surface electric field from the localized surface plasmon resonance (LSPR) of Cu nanoparticles further reduces energy barriers for redox reactions, improving seawater splitting activity. This work provides valuable insights into intergrating the reaction environment, broad solar absorption, LSPR, and active single atoms into a core‐shell photocatalyst design for efficient and robust solar‐driven seawater splitting.

## Graphical abstract

A core‐shell photocatalyst comprising atomic‐dispersed Co and plasmonic Cu nanoparticles is developed for efficient photocatalytic hydrogen production from seawater. This photocatalyst achieves strong synergy between the reaction environment, broad light absorption, plasmonic electric field, and active atomic sites, thereby enabling optimal seawater hydrogen production through redox reactions.

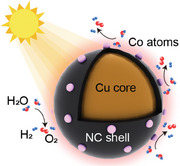

## 1 Introduction

Amidst an escalating global demand for clean energy in response to climate change, the imperative for advancing hydrogen energy production technologies is increasingly apparent.^[^ ^1^ ^]^ Within the contemporary landscape of energy conversion technologies, photocatalysis stands out as an energy‐saving and environmental friendliness with capability of utilizing abundant resources, such as sunlight and water, to generate hydrogen.^[^ ^2^, ^3^ ^]^ Additionally, attention is progressively shifting toward the utilization of seawater for photocatalytic hydrogen production. Seawater is an earth‐abundant and virtually limitless resource, covering ≈71% of the Earth's surface, and providing 96.5% of the planet's water resource for sustainable energy generation.^[^ ^4^ ^]^ Leveraging the immense expanse of seawater as a feedstock for hydrogen production via photocatalysis has far‐reaching potential applications.^[^ ^5^ ^]^ Developing photocatalysts with high hydrogen production performance and stability under saline environments is essential for the development of scalable photocatalytic systems. The utilization of untreated water sources, notably seawater, to drive photocatalytic reactions is on the rise. However, achieving higher reaction efficiency and long‐term stability demands elevated criteria for photocatalysts. This necessitates the integration of co‐catalysts,^[^ ^6^ ^]^ heterojunctions,^[^ ^7^ ^]^ and other engineering strategies,^[^ ^8^ ^]^ although their practicality remains subject to scrutiny.

Despite yielding promising research outcomes, the efficient production of hydrogen through seawater splitting largely relies on noble metals and/or sacrificial agents.^[^ ^2^, ^9^, ^10^, ^11^ ^]^ The scarcity of noble metals and the additional costs associated with sacrificial agents have limited their widespread application due to high expenses. Hence, there is an urgent need to develop low‐cost, high‐performance catalysts without noble metals as co‐catalysts or sacrificial agents. Atomically dispersed non‐noble metal atoms immobilized on nitrogen‐doped carbon materials (M‐NC) are widely recognized for their efficiency as catalysts in photocatalytic reactions, including carbon dioxide (CO_2_) reduction,^[^ ^12^ ^]^ H_2_O_2_ production,^[^ ^13^ ^]^ CH_4_ oxidation,^[^ ^14^ ^]^ and water splitting,^[^ ^15^ ^]^ etc. Their high performance stems from the abundance of highly exposed active sites and efficient transport of photogenerated charge carriers. By modulating the coordination environment to modify the electronic structure of atomic metal sites, M‐NC can enhance activity, selectivity, and reaction kinetics for specific catalytic reactions. Besides, metal plasmonic nanoparticles (NPs) with localized surface plasmon resonance (LSPR) effects have shown great potential in energy conversion due to their ability to expand the photo‐response range,^[^ ^16^, ^17^ ^]^ and activate chemical bonds through the LSPR‐induced electric‐field (E‐field).^[^ ^18^, ^19^ ^]^ In typical plasmonic photocatalytic systems, plasmonic NPs are usually deposited onto functional semiconductors.^[^ ^20^, ^21^, ^22^ ^]^ However, this configuration limits plasmonic enhancement to the interface region between the metal and semiconductor, and also causes the plasmonic NPs to potentially block reaction sites on the semiconductors. A core‐shell structure with the plasmonic material at the core and the semiconductor material at the shell could overcome these challenges by uniformly distributing plasmonic enhancement around the NPs without blocking reaction sites of the semiconductor.^[^ ^23^ ^]^ However, to our best knowledge, few studies have reported on the design of core‐shell photocatalysts that combine M‐NCs with the LSPR effect specifically for seawater splitting.

Herein, we developed a photocatalyst with a core‐shell structure named Co─NC@Cu, comprising Cu NPs as cores covered with N‐doped carbon (NC) as a shell, anchored with Co atoms, for photocatalytic seawater splitting. This innovative design aims to enhance photocatalytic activity and stability by leveraging the synergistic effects of the core‐shell structure and the properties of the individual components. The stable carbon shell can inhibit the oxidation of Cu in seawater, thereby preventing photocatalyst degradation in the reaction environment. As the core, Cu nanoparticles not only exhibit broad‐spectrum light absorption but also concentrate light energy through the LSPR effect, generating and providing hot plasmonic charge carriers to the Co─NC catalyst, thereby facilitating the redox reactions on its surface. Atomically dispersed Co in the NC serves as effective active reaction sites, supported by Density Functional Theory (DFT) calculations, and exhibits low reduction reaction energy barriers. Furthermore, the local E‐field generated by the core‐shell NPs reduces the reduction and oxidation reaction energy barriers, thereby enhancing the overall redox reaction activity. This photocatalyst demonstrates superior performance and long‐term stability compared to previously reported systems (Table S1, Supporting Information). This work provides guidance for achieving optimal seawater hydrogen production through efficient coupling of the reaction environment, broad light absorption, plasmonic E‐field, and active atomic sites. We believe these findings contribute to the development of highly efficient photocatalysts for sustainable energy solutions.

## 2 Characterization of Co─NC@Cu Catalysts

**Figure 1:**
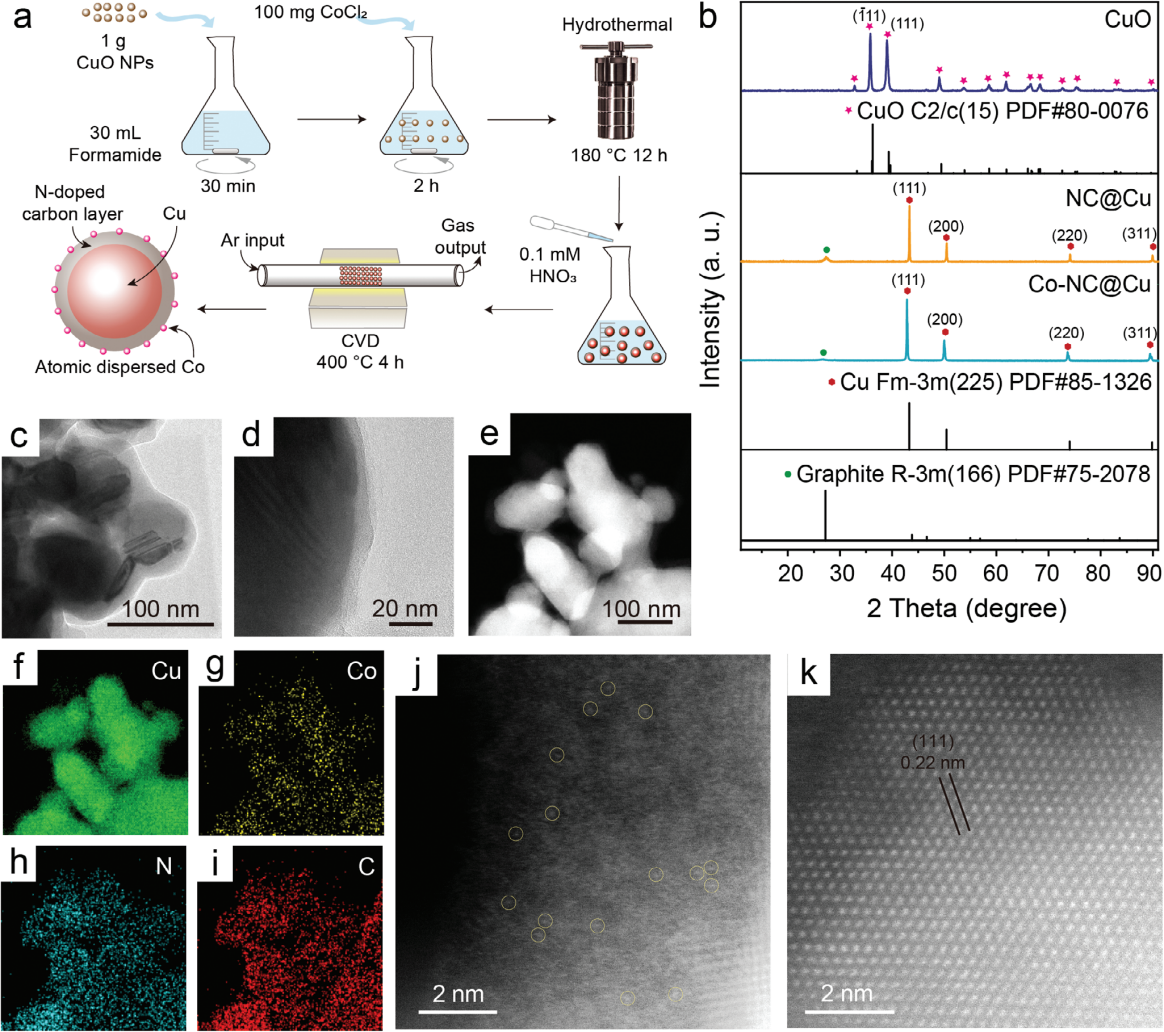
Synthesis, composition and morphology of Co─NC@Cu. a) Schematic diagram of the synthesis process of Co─NC@Cu. b) XRD patterns of CuO, NC@Cu, Co─NC@Cu and the corresponding standard PDF cards. c) TEM images of Co─NC@Cu and d) zoomed‐in image of a Cu NP edge. e) The HADDF‐STEM and EDS element mapping of Co─NC@Cu for f) Cu, g) Co, h) N, and i) C. The AC HADDF‐STEM local images of j) Co single atoms anchored N‐doped carbon shell (i.e., Co─NC), and k) the core Cu.

The synthesis method^[^ ^14^ ^]^ for the Co─NC@Cu catalyst is illustrated in **Figure** **1**a, where CuO and CoCl_2_ are dispersed in formamide (HCONH_2_) and subjected to hydrothermal treatment at 180 °C for 12 h. Under these reaction conditions, formamide underwent conversion to the amorphous nitrogen‐doped carbon (NC) material. After dilute acid treatment and Ar annealing, the Co precursor formed a Co─N bond with the NC layer. The Cu NPs are reduced from the initial CuO NPs during this process to form a core. Therefore, a core‐shell structure has been proposed, comprising a Co─N coordination‐based Co─NC shell covering the Cu NPs core. The X‐ray diffraction (XRD) results (Figure 1b) reveal that the main diffraction peaks of the core component for both NC@Cu and Co─NC@Cu samples match with face centered cubic Cu (Figure 1b), without any apparent peaks corresponding to CuO substances. This implied that the initial CuO NPs were successfully reduced to Cu NPs. Furthermore, the XRD patterns of NC@Cu and Co─NC@Cu showed a weak C peak, which is associated with the NC shell layer. Transmission electron microscopy (TEM) images (Figure 1c,d) depicted the surface of Cu NPs was covered by thin NC layer. To further determine the dispersion and position of Co atoms, we characterized the Co─NC@Cu on an aberration‐corrected TEM (AC‐TEM), using high‐angle annular dark‐field scanning TEM (HAADF‐STEM) coupled with energy‐dispersive X‐ray spectroscopy (EDS) elemental mapping (Figure 1e–i; Figure S1, Supporting Information) and atomic‐resolution HAADF‐STEM imaging (Figure 1j). These results further affirmed the dispersion of Co atoms on the NC layer, with Co atoms appearing as isolated bright spots (as indicated by the yellow circles in Figure 1j). No Co nanoparticles or clusters were observed. The inductively coupled plasma optical emission spectrometry (ICP‐OES) analysis revealed that the actual Co loading was 0.40 wt.% (Table S2, Supporting Information). Highly crystalline Cu lattices of the face‐centered cubic (fcc) structure is evident in the atomic‐resolution STEM image shown in Figure 1k and Figure S2 (Supporting Information).^[^ ^24^ ^]^ After long‐term (343 h) stability tests, the characteristic XRD peaks of Cu and C in Figure S3 (Supporting Information) remain comparable to the pristine Co─NC@Cu sample (Figure 1b). The lack of Cu core's oxidation after prolonged seawater splitting (Figure S4, Supporting Information) indicates the NC shell's effective protective attributes. The EDS elemental mapping results (Figure S5, Supporting Information) confirm that the sample maintained the presence of constituent elements, notably Co, which remains well‐dispersed throughout the material without any observable signs of aggregation.

**Figure 2:**
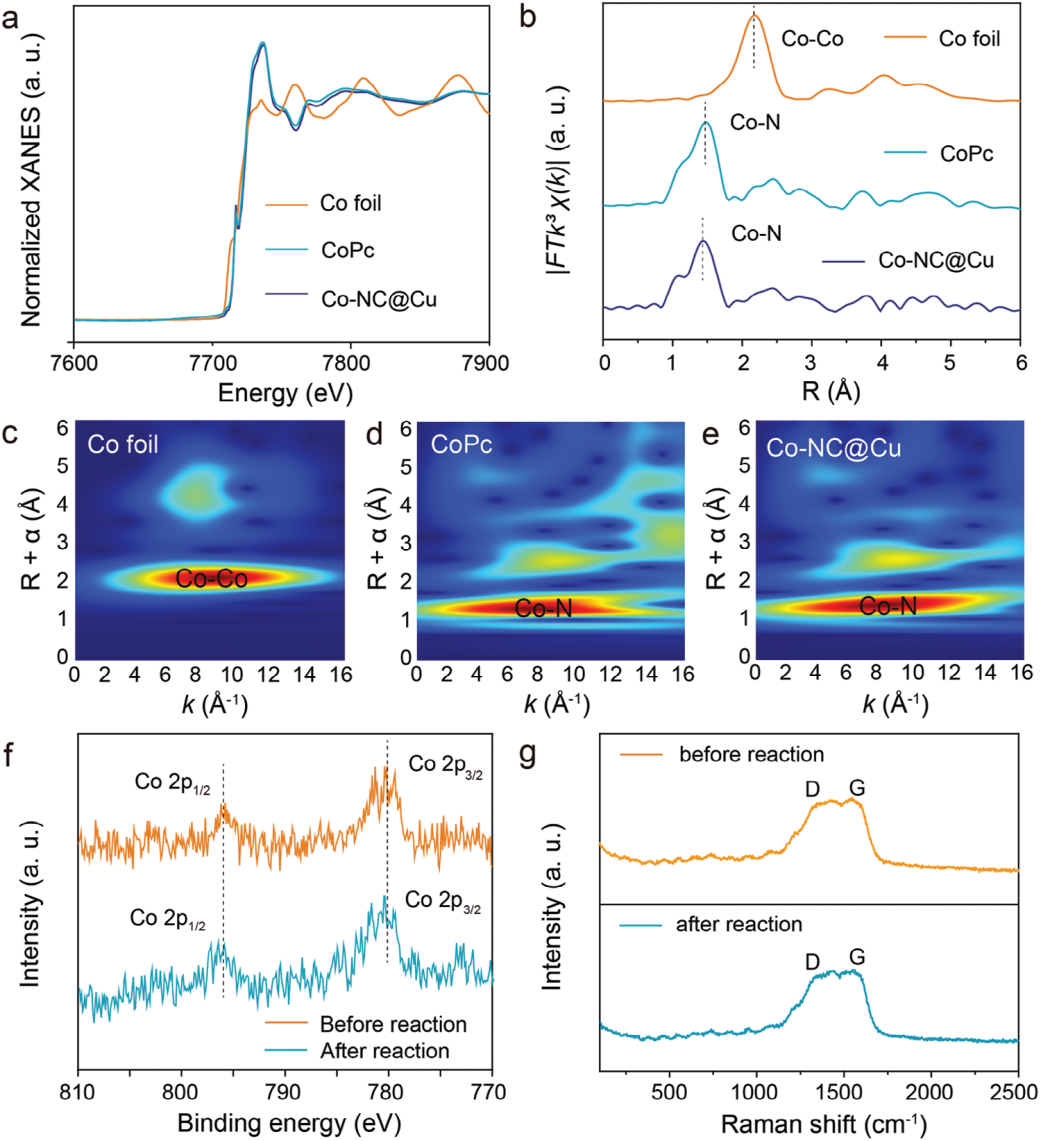
a) The XANES and b) FT‐EXAFS of Co K‐edge for Co foil, CoPc and Co─NC@Cu. WT‐EXAFS for c) Co foil, d) CoPc and e) Co─NC@Cu. f) XPS spectra for Co 2p. g) Raman spectra for Co─NC@Cu before and after 343 h reaction.

X‐ray absorption spectroscopy (XAS) analysis was conducted to investigate the coordination environment of Co, providing further confirmation of the atomic dispersed Co in Co─NC@Cu and offering deeper insights into the catalyst structure. The acquired data were compared with Co foil and Cobalt(II) phthalocyanine (CoPc) as reference materials. The normalized X‐ray absorption near‐edge structure (XANES) spectra of the Co K‐edge is depicted in **Figure** **2**a. As indicated by the XANES spectra, the comparison of near‐edge adsorption energy between Co─NC@Cu, CoPc, and Co foil, indicates that the Co K‐edge energy of Co─NC@Cu is comparable with that of CoPc. This result suggests the similar chemical states of Co between Co─NC@Cu and CoPc. In addition, Co─NC@Cu exhibits similar pre‐edge profile to that of CoPc for Fourier‐transform extended X‐ray absorption fine structure (FT‐EXAFS) spectra in Figure 2b. In the FT‐EXAFS Co K‐edge, a prominent peak at ≈1.43 Å corresponds to the first‐shell coordination of the Co─N bond,^[^ ^25^ ^]^ mirroring the contribution observed in the CoPc reference value. Notably, no discernible Co‐Co peak was detected at ≈2.17 Å, indicating the negligible presence of metallic Co species.^[^ ^26^ ^]^ These findings corroborated the atomic dispersion of the Co─N coordination in Co─NC@Cu, aligning with the results of Co atomic dispersion observed in the HAADF‐STEM image as discussed above. The wavelet transform EXAFS (WT‐EXAFS) spectra (Figure 2c) illustrated that Co foil exhibited distinct sharp regions, representing Co‐Co metal bonds.^[^ ^27^ ^]^ The contour plots for Co─NC@Cu in Figure 2d and CoPc in Figure 2e exhibit similar features, further supporting the formation of Co─N coordination in the Co─NC@Cu catalyst. Additionally, X‐ray photoelectron spectroscopy (XPS) in Figure 2f and Figures S6 and S7 (Supporting Information) was employed to investigate the valence states of elements. The Co 2p spectra of Co─NC@Cu exhibited two characteristic main peaks at 796.0 and 780.3 eV corresponding to the Co 2p3/2 and Co 2p1/2 orbitals before and after reaction.^[^ ^28^ ^]^ The peaks of N1s corresponding to graphite N (401.1 eV), pyridinic N (398.6 eV), pyrrolic N (400.1 eV), and N‐metal species (399.4 eV, corresponding to Co─N interaction) can be resolved in N 1s XPS spectra (Figure S7, Supporting Information).^[^ ^29^ ^]^ Furthermore, Raman spectroscopy confirmed the presence of graphitic carbon structure. The D band at 1433 cm^−1^ is attributed to in‐plane vibrations of sp_2_ carbon atoms in graphite structure, while the G band at 1678 cm^−1^ originates from defects in graphene.^[^ ^30^ ^]^ The electron paramagnetic resonance (EPR, Figure S8, Supporting Information) spectrum revealed the presence of N defects (i.e., the doped N) in Co─NC@Cu. The observed signal at g = 2.003 confirmed these N defects in the sample, suggesting atomic‐level coordiation of Co atoms with N within NC@Cu.^[^ ^31^ ^]^ The above results have proved Co single atoms were successfully coordinated with N rather than forming the NPs.

**Figure 3:**
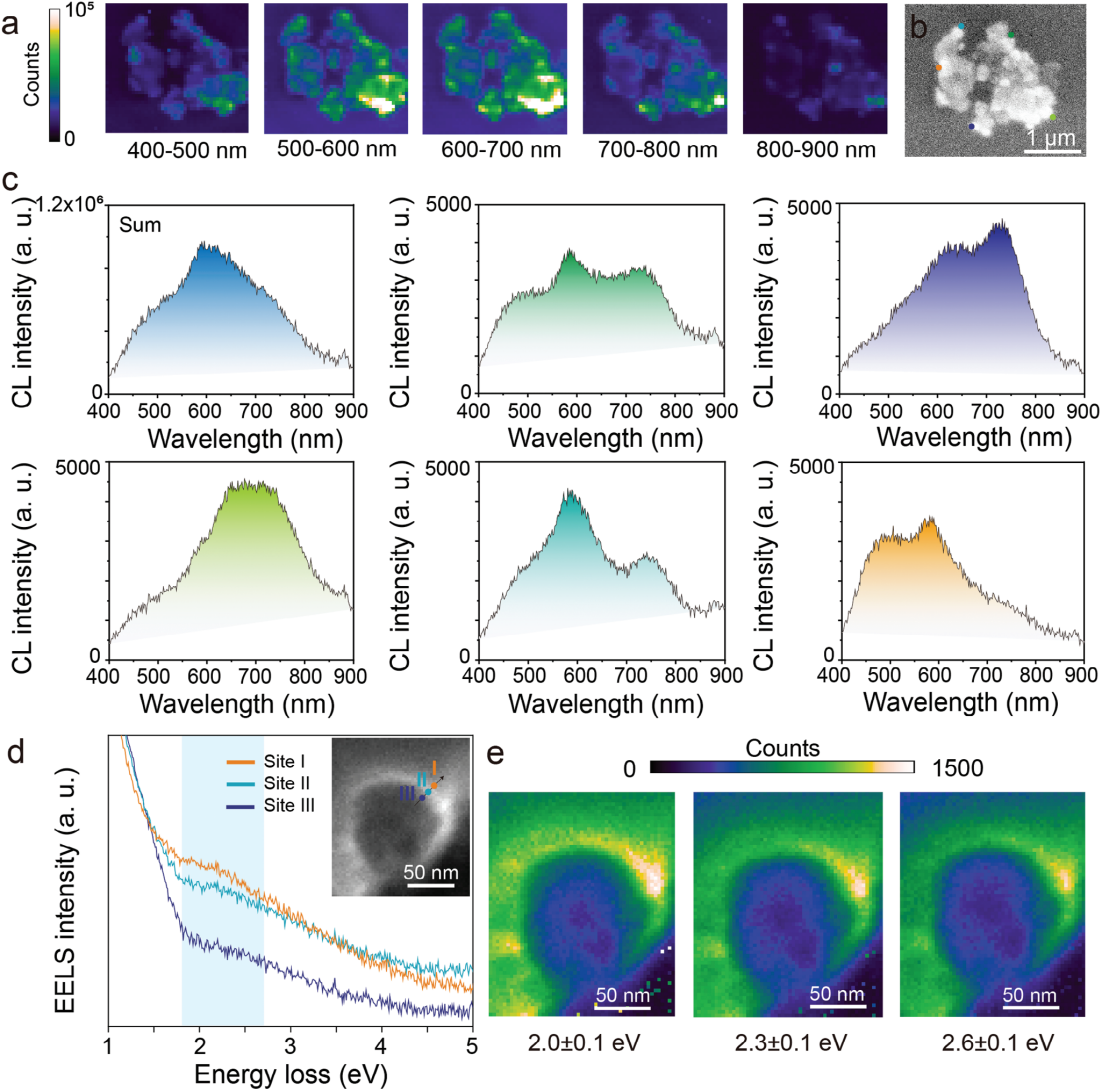
a) CL intensity maps integrated across different wavelength ranges from 400 to 900 nm. b) CL emission signal for Co─NC@Cu with the corresponding scanning electron microscopy (SEM) image. c) CL emission full/Sum spectrum (blue) and the spectra at the different sites indicated in b) with the corresponding colors. d) EELS spectra obtained from sites I, II, and III as indicated in the inset image. e) EELS mapping of Co─NC@Cu NPs at 2.0 ± 0.1, 2.3 ± 0.1, and 2.6 ± 0.1 eV, corresponding to the blue‐shaded peak region in the EELS spectra in d).

The spatial and energy distribution of the resonances in Co─NC@Cu was investigated using spatially resolved cathodoluminescence (CL) spectroscopy. The CL signal was analyzed by integrating it across different wavelength ranges from 400 to 900 nm. **Figure** **3**a illustrates the intensity maps obtained by integrating the CL emission across different wavelength ranges. The core‐shell NPs with varied particle sizes (Figure S9, Supporting Information) results in broadband absorption in the visible and near‐infrared regions. This phenomenon arises from the existence of multiple plasmon modes excited within the differently sized plasmonic NPs.^[^ ^32^ ^]^ Figure 3b distinctly depicts the CL emission signal reflected from the brightened regions, indicating that the area of strong CL signal covers multiple nanoparticles. CL measurements were conducted in raster scanning mode, locally exciting the nanostructure, and acquiring the full spectra of each pixel of the scanned image as shown in Figure 3b. The CL spectra from different regions of the sample are represented in the sub‐figure of Figure 3c and Figures S10 and S11 (Supporting Information). The analysis of the CL signals at different edge sites (shown with different colors in Figure 3b) revealed one or more peaks in the spectral region of 500–750 nm, where the signals exhibit relatively stronger intensity. This analysis further highlights the generation of multiple resonances at different wavelengths by the different sized Cu cores in the Co─NC@Cu catalyst sample.^[^ ^33^ ^]^ The multiple resonances contribute to an overall broadband absorption in the visible and near‐infrared spectra.^[^ ^34^ ^]^

To gain insights into the enhancement of optical absorption by plasmonic Cu NPs, electron energy loss spectroscopy (EELS) was coupled with STEM‐HAADF imaging to measure the local optical response.^[^ ^35^ ^]^ In Figure 3d and Figures S12 and S13 (Supporting Information), the EELS spectra of Co─NC@Cu particles is depicted, exhibiting a distinctive absorption shoulder. In the line scan, optical absorption in the range of 1.8–2.7 eV is depicted, corresponding to wavelengths between 459 and 689 nm (illustrated in the blue‐shaded region in Figure 3d). This range matches the complete Sum spectra of CL presented in Figure 3c and Figure S10 (Supporting Information). Note that in Figure 3d, the intensity at position I (near the edge of the NC shell) is higher than that at position II (within the NC shell), both of which are further higher than the intensity at position III (within the copper core). These results, combined with the EELS maps in Figure 3e, confirm the LSPR effect in the Co─NC@Cu NPs, which is well‐distributed around the Co─NC@Cu NPs without quenching. The above results highlight that Co─NC@Cu NPs possess the capability to absorb broad light from the visible to near‐infrared regions, endowing this core‐shell structure with enhanced light absorption across a wider wavelength range.^[^ ^6^ ^]^ The combined CL and EELS studies presented here provide robust evidence for the LSPR phenomenon in Co─NC@Cu and its broad light harvesting capabilities.

## 3 Seawater Splitting Performance of Co─NC@Cu Catalysts

**Figure 4:**
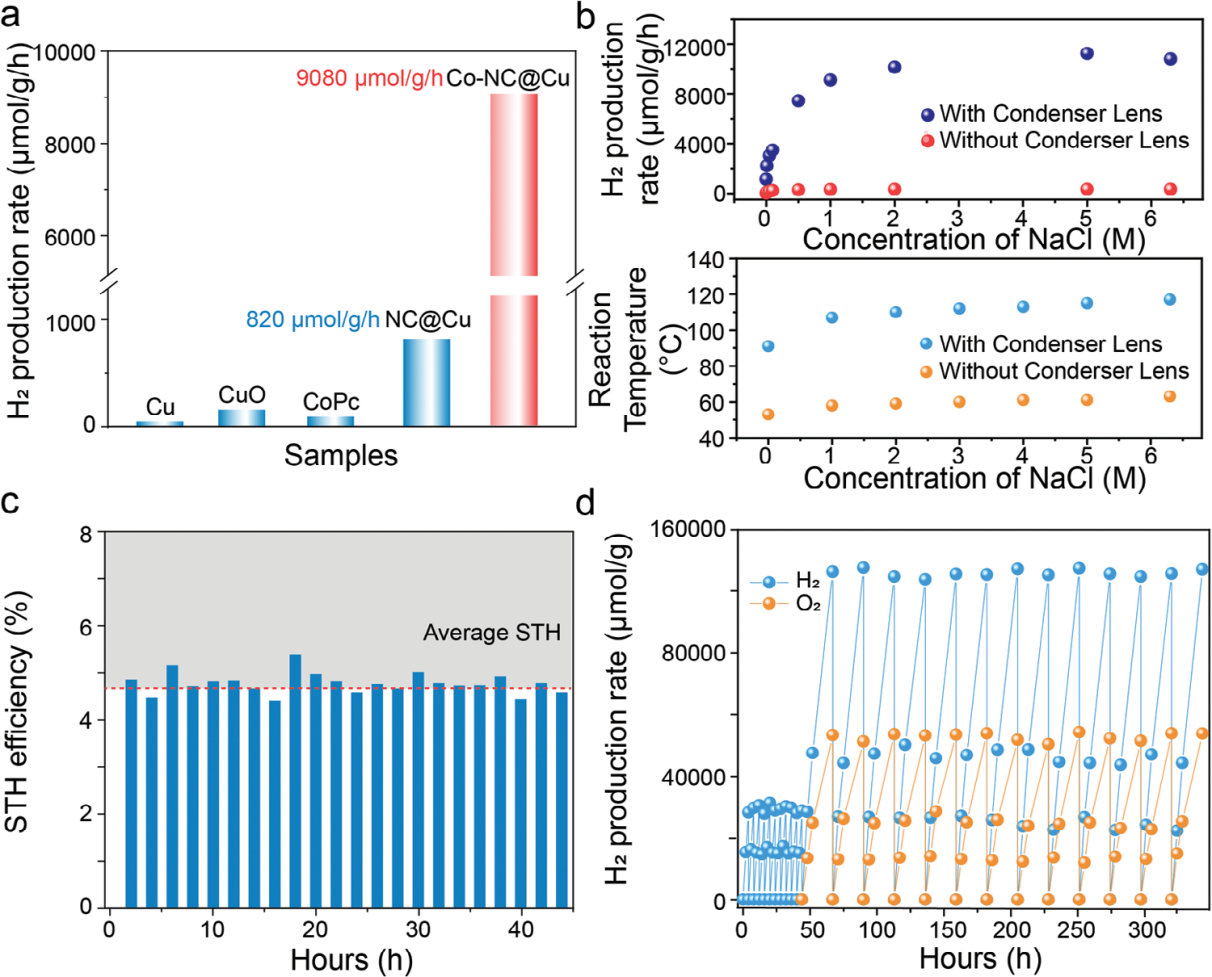
a) Comparison of the H_2_ production rate from light‐driven seawater splitting between Co─NC@Cu and its control samples. b) The H_2_ production rate and reaction temperature as dependent on the NaCl concentration. c) STH efficiency recorded every 2 h in the first 44 h reaction. d) Long‐term stability tests up to 343 h.

To comprehensively study the light‐driven seawater splitting performance of the synthesized Co─NC@Cu, the Xenon light source equipped with a condenser lens is utilized, which generated a spot with a diameter of 1.6 cm and a light intensity of 600 mW·cm^−2^ (AM 1.5G). Notably, no sacrificial reagents and external heat source were used throughout the process. Photocatalytic seawater splitting performance of different samples was compared using gas products collected by gas chromatography (GC), as depicted in **Figure** **4**a. Notably, bare Cu, CuO and CoPc consistenly show poor hydrogen production for photocatalytic seawater splitting. In contrast, Co─NC@Cu with Co single atoms anchored on the NC shell outperforms NC@Cu without Co single atoms by over ten times. Accordingly, by controlling different CoCl_2_ precursors during the synthesis process to optimize the Co loading (Figure S14, Supporting Information), the optimized Co─NC@Cu sample achieves the highest H_2_ production rate of 9080 µmol g^−1^h^−1^ in 1 m NaCl seawater, demonstrating advanced performance in a noble metal‐free, sacrificial agent‐free photocatalytic seawater splitting system (Table S1, Supporting Information).

To explore the potential roles of light and heat in the seawater splitting, additional experiments were conducted (Figure S15, Supporting Information). The results indicated that only low‐yield H_2_ products were obtained under unfocused normal solar illumination, while no H_2_ or O_2_ products were detected when we solely heated the catalysts up to 150 °C. Additional reference experiments indicate that, when controlling for the same enhanced incident light/photons, the reaction environment temperature significantly influences H_2_ generation (Figure S16a, Supporting Information). This suggests that the thermal effect contributes to H_2_ production, rather than the increase in H_2_ generation being solely due to the enhanced incident light/photons. Additionally, the relationship between H_2_ yield and wavelength of light was investigated (see Figure S16b, Supporting Information). The results indicate that the correlation between H_2_ yield and wavelength aligns with the results from CL spectroscopy and EELS, illustrating that the optical absorption in the range of 459–689 nm corresponds to the highest H_2_ production rates. While the catalyst can absorb a significant amount of 700 nm photons, the lower energy of these photons results in photo‐excited electron‐hole pairs with lower energy, which have relatively lower reduction/oxidation potentials. Consequently, this contributes less to the photocatalytic process. These results imply that the photocatalytic reaction is driven by a combination of broad‐spectrum solar illumination and the light‐focusing induced photothermal effect, rather than thermal or solar input alone. The photothermal effect includes both photo‐induced hot carrier generation^[^ ^36^ ^]^ and reaction environment heating.^[^ ^37^ ^]^ The reaction‐environment heating results in an increase in the thermal conductivity of seawater, which may in turn enhance heat transport, thereby promoting photothermal conversion efficiency.^[^ ^38^, ^39^ ^]^ Collectively, these factors enhance the photoreaction and increase the rate of H_2_ generation.^[^ ^9^ ^]^ Moreover, the effect of NaCl concentration on H_2_ production rate and reaction temperature was studied (Figure 4b). As NaCl concentration increases from 0 to 1 M, the temperature rises to ≈110 °C (bottom sub‐figure), accompanying an increase in H_2_ production rate to 9080 µmol g^−1^h^−1^ (top sub‐figure). Further increasing NaCl concentration only slightly impacts temperature and H_2_ production, eventually reaching saturation.

Time‐resolved photoluminescence (TRPL) measurements (Figure S17, Supporting Information) and DFT calculations (Figures S18–S21, Supporting Information) were further employed to study the effect of NaCl content on photocatalytic seawater splitting. The DFT analysis shows that traditionally inert cations/anions in seawater, such as Cl^−^, Na^+^, as well as their hydrated forms, adsorb onto the surface of Co─NC@Cu, inducing charge polarization, as shown in Figures S18–S21 (Supporting Information). The fitting of the TRPL spectra revealed an increase in carrier lifetime (Table S3, Supporting Information), which can be attributed to the suppression of charge recombination due to the local polarization effect introduced near the surface by the adsorbed ionic species.^[^ ^2^ ^]^ Therefore, the saline content in seawater can significantly enhance the performance of thermal‐assisted photocatalytic seawater splitting by inducing strong charge polarization, and thereby prolonging carrier lifetimes.^[^ ^2^ ^]^

By recording the performance tests every 2 h during the first 44 h reaction, the received average solar‐to‐hydrogen (STH) conversion efficiency reaches 4.78% (Figure 4c). The Co─NC@Cu sample also exhibits remarkable long‐term stability and consistent photoactivity over 340 h (Figure 4d), with the yield of H_2_ and O_2_ remaining close to the stoichiometric 2:1 ratio, demonstrating its overall water splitting capability and long‐term stability. The STH result and long‐term stability of Co─NC@Cu surpass most reported literature (Table S1, Supporting Information).

## 4 Theoretical Calculations

**Figure 5:**
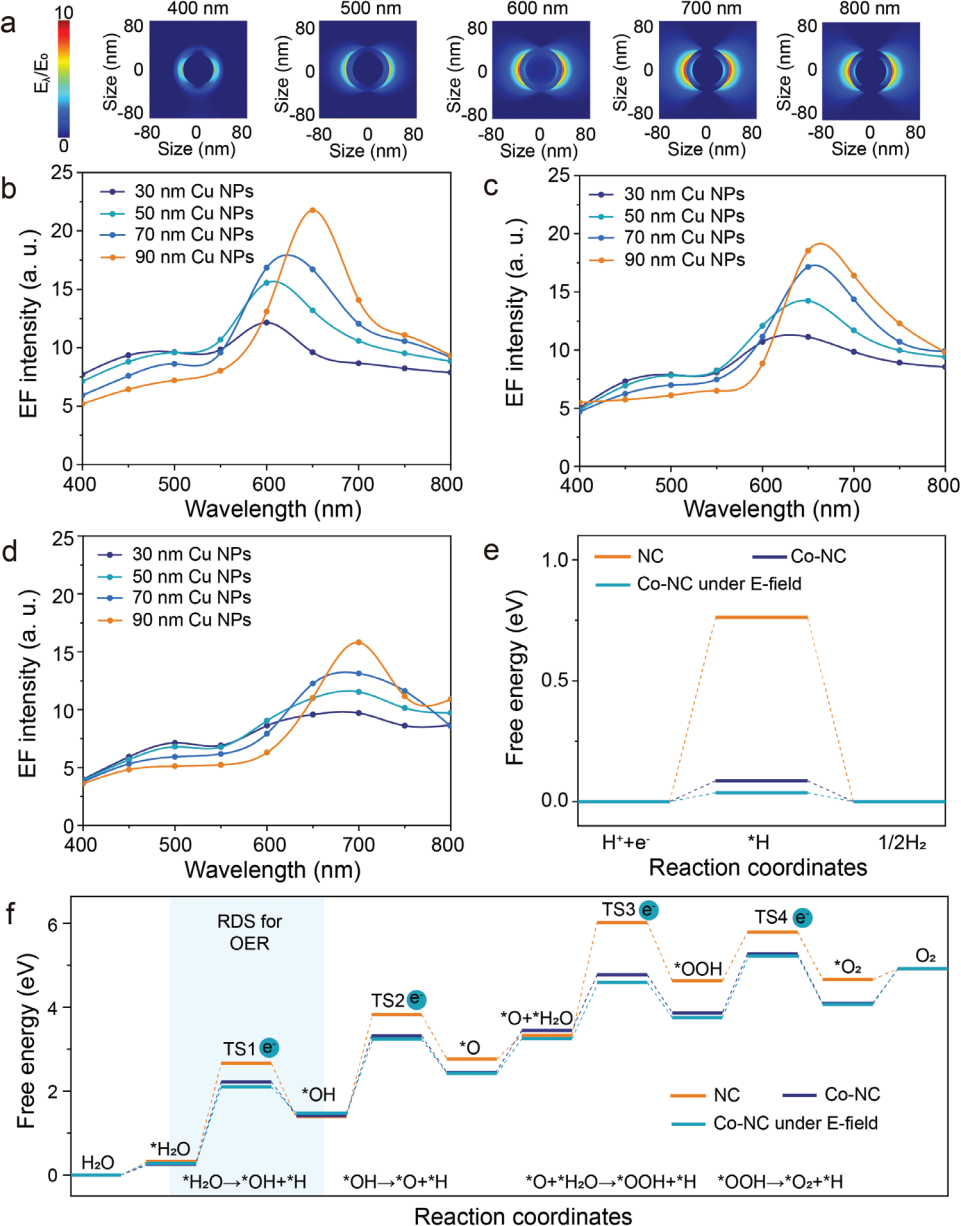
a) The simulated E‐field distribution of Co─NC@Cu under light irradiation at different wavelength ranges using FDTD simulations. b) The maximum E‐field (EF) intensity versus. incident light wavelengths for Cu NPs with different size. c) The maximum E‐field intensity versus. incident light wavelengths for Co─NC@Cu with c) 5 nm and d) 10 nm shell thickness. The E‐field intensity of the incident light was set to 1 V/m, and the incident light intensity was set to 600 mW cm^−2^. Calculated Gibbs free energy of e) HER and f) OER on Co─NC@Cu, with E‐field perturbation included if mentioned. Four transition state (TS) processes include TS1 as *H_2_O → *OH + *H, TS2 as *OH → *O + *H, TS3 as *O + *H_2_O → *OOH + *H, and TS4 as *OOH → *O_2_ + *H.

To gain deeper insights into the local E‐field distribution associated with the plasmonic properties of Co─NC@Cu, DFT calculations were conducted to predict optical properties (Figures S22–S24, Supporting Information) and compared to Finite Difference Time Domain (FDTD) simulations were conducted by structural modelling based on the STEM‐HAADF results. As depicted in **Figure** **5**a–d and Figures S25–S27 (Supporting Information), FDTD simulation offers more information of the local E‐field enhancement induced by the LSPR effect across the entire Co─NC@Cu system. Figure 5a illustrates the simulated local E‐field distribution of Co─NC@Cu with a 50 nm Cu NP core and 10 nm NC shell at five different wavelengths. The E‐field enhancement is particularly prominent from the visible and near‐infrared range, with their negligible quenching phenomenon observed on the thin Co─NC shell. The characteristics of the local electromagnetic field arising from the plasmon resonance of the material surface in the core‐shell structure can be affected by performing different radius of the metallic core or the thickness of the carbon shell.^[^ ^35^ ^]^ As evidenced by the spectra in Figure 5b–d, enlarging the size of the Cu NPs core results in a red shift of the dominated resonant peaks. The increase in the core size of Cu NPs forms a larger plasmon resonator, thereby reducing the plasmon frequency and energy. Figure 5c,d demonstrate that increasing the Co─NC shell thickness (5 and 10 nm) also leads to a minor red shift in peak energy and a slight decrease in E‐field intensity, while still maintaining over ×10 local E‐field enhancement when Cu NP size exceeds 30 nm under full‐spectrum solar illumination. Therefore, the enhanced local E‐field of the Cu NP core effectively transfers to the Co─NC shell surface without significant quenching by the shell.

The local E‐field profoundly influences the desorption and adsorption of intermediates during the reaction steps, thereby activating the chemical bonds and energy of the intermediates and subsequently modulating the Gibbs free energy and reaction activity. The previous studies have validated that plasmonic E‐fields can thermodynamically regulate reaction pathways.^[^ ^21^ ^]^ As depicted in Figure 5e,f; Figures S28 and S29 and Table S4 (Supporting Information), our investigation examines the effect of the E‐field on the Gibbs free energy of each reaction step for NC shells with or without Co atoms as catalytic reaction sites. HER (hydrogen evolution reaction) is typically described as a three‐step process: the initial state H^+^ + e^−^, the intermediate adsorbed H^*^, and the free energy of the final product ½H_2_, and the ΔG_H_^*^ is considered to describe the activity.^[^ ^40^, ^41^, ^42^ ^]^ Co─NC were found to be more thermodynamically favourable for H_2_ production compared to the sole NC shown in Figure 5e. This provides a foundation for prioritizing the design of catalysts with Co─N coordination as ideal water‐splitting catalysts. It is worth noting here that the applied E‐field further reduces the ΔG_H_^*^ for Co─NC under E‐field perturbation.

In the DFT model described in Figure 5f, which involves the oxygen evolution reaction (OER) mechanism involving four concerted proton‐electron transfer steps, we investigated the impact of the E‐field on the Gibbs free energy of each reaction step in the OER. The reaction barriers and rate‐determining steps (RDS) are crucial descriptors for assessing OER performance, and we modeled the intermediate adsorption structures for all potential sites in Figure S29 (Supporting Information). The free energy reaction pathway analysis indicates that Co─NC exhibits better oxidation reaction activity compared to NC alone. This is due to the lower free energy barrier of the OER process for Co─NC compared to NC from a thermodynamic perspective, as shown in Figure 5f. Furthermore, transition state calculations reveal that the ^*^H_2_O → ^*^OH+^*^H step is the RDS (TS1). Although the change in Gibbs free energy of Co─NC during OER reaction steps under E‐field perturbation is slight, it is noteworthy that the energy barrier of RDS decreases from 1.96 eV under no E‐field to 1.83 eV under ×10 E‐field perturbation, which favors the activity of the oxidation reaction. Therefore, the NC with Co single atoms was found to exhibit thermodynamically favorable OER under E‐field. The calculations show perturbing E‐fields play a crucial role in achieving ideal photocatalytic water splitting performance, particularly for the LSPR‐active Co─NC@Cu core‐shell. Besides, although FDTD simulations reveal that Co─NC nanoparticles maintain relatively high E‐field intensity at a wavelength of 700 nm (Figure 5b–d), the photon energy at this wavelength is relatively low. Therefore, the E‐field perturbation alone does not determine H₂ performance. Instead, under the local electric field perturbation induced by LSPR, the energy barriers for reduction and oxidation reactions are reduced, and their enhancement degree on the photocatalytic activity is affected by the incident photon energy (i.e., light wavelength). This accounts for the observed experimental data where the relatively high E‐field intensity at 700 nm does not translate into enhanced H₂ production due to lower photon energy and decreased effectiveness of photo‐excited carriers in driving the redox reactions.

## 5 Conclusion

In conclusion, we present a novel atomic Co dispersed on an NC@Cu core‐shell structure catalyst for efficient photocatalytic seawater splitting to generate H_2_, without requiring sacrificial agents or noble metal co‐catalysts. Comprehensive characterization confirms the successful synthesis of the core‐shell structure with Co single atoms. Reaction environment analysis reveals that the focus‐light‐driven thermal effects and salt‐ion‐induced charge polarization contribute to enhancing photocatalytic performance. Further investigations demonstrate that this catalyst exhibits broad‐spectrum light absorption ability, facilitating increased participation of photogenerated charge carriers in reactions. Besides, the LSPR effect originated from the core Cu NPs in Co─NC@Cu is not quenched by the embraced NC shell. Theoretical calculations support that the Co─NC single atoms provide ideal active sites for water splitting, with reduced energy barriers for both reduction and oxidation reactions under the local E‐field perturbation induced by LSPR, further boosting photocatalytic activity. Consequently, Co─NC@Cu achieves high STH efficiency with long‐term stability in seawater splitting for hydrogen production. This presents a promising strategy utilizing non‐noble metal plasmonic NPs for efficient solar‐driven hydrogen generation from abundant saline resources. The insights from this study will be useful for developing other noble metal‐free, sacrificial agent‐free, single‐atom based and plasmonic photocatalysts.

## Conflict of Interest

The authors declare no conflict of interest.

## Supporting information

Supporting Information

## Data Availability

The data that support the findings of this study are available from the corresponding author upon reasonable request.
