# Supplementary material for: Atomic Dispersed Co on NC@Cu Core‐Shells for Solar Seawater Splitting
Source: Adv Mater. 2024 Oct 14;36(49):2406088. doi: 10.1002/adma.202406088 (PMC11619220; doi:10.1002/adma.202406088)
Supplement: Supplementary file 1 — Supporting Information [file ADMA-36-2406088-s001.docx]

Supporting Information

Atomic Dispersed Co on NC@Cu Core-Shells for Solar Seawater Splitting

Zhehao Sun, Shuwen Cheng, Xuechen Jing, Kaili Liu, Yi-Lun Chen, Ary Anggara Wibowo, Hang Yin, Muhammad Usman, Daniel MacDonald, Soshan Cheong, Richard F. Webster, Lucy Gloag, Nicholas Cox, Richard D. Tilley and Zongyou Yin*

Z. Sun, S. Cheng, X. Jing, K. Liu, Y.-L. Chen, H. Yin, M. Usman, L. Gloag, N. Cox, Z. Yin

Research School of Chemistry, The Australian National University; Canberra, Australian Capital Territory, 2601, Australia.
E-mail: zongyou.yin@anu.edu.au

A. A. Wibowo, D. MacDonald

School of Engineering, The Australian National University, Canberra, Australian Capital Territory, 2601, Australia

S. Cheong, R. F. Webster, R. D. Tilley

Electron Microscope Unit, Mark Wainwright Analytical Centre, University of New South Wales, Sydney, New South Wales, 2023, Australia

**Table of Contents**

Supporting methods................................................................................................................................S3

Figure S1. The HADDF-STEM and EDS element mapping...................................................................S8

Figure S2. The AC HADDF-STEM local images of core Cu............................................................... S8

Figure S3. The XRD patterns of Co-NC@Cu after the reaction............................................................S9

Figure S4. XPS spectra for Cu...............................................................................................................S9

Figure S5. The HADDF-STEM and EDS element mapping.................................................................S10

Figure S6-7. XPS spectra for C and N...................................................................................................S10

Figure S8. EPR spectra..........................................................................................................................S11

Figure S9. Size distribution histogram..................................................................................................S11

Figure S10. Panchromatic CL intensity map.........................................................................................S12

Figure S11. CL emission spectra at the different sites...........................................................................S12

Figure S12. Fitting of line scan for EELS results.................................................................................S13

Figure S13. Plasmon signal mapping....................................................................................................S13

Figure S14-16. The additional experiments..........................................................................................S14

Figure S17. TRPL spectra....................................................................................................................S15

Figure S18-21. Calculated Bader charges and charge density difference .............................................S16

Figure S22-24. Refractive index (n) and extinction co-efficient (k).....................................................S18

Figure S25-27. The simulated E-field distribution................................................................................S20

Figure S28. DFT modeling....................................................................................................................S23

Figure S29. The optimized structure with intermediates adsorption.....................................................S23

Table S1. Summary of photocatalytic hydrogen production using seawater.........................................S24

Table S2. The ICP analysis...................................................................................................................S24

Table S3. The fitting of TRPL about carrier lifetime.............................................................................S25

Table S4. The relevant DFT calculation results....................................................................................S25

Author Contributions............................................................................................................................S25

Reference.….........................................................................................................................................S26

**Supporting methods**

**Chemicals preparation.** CuO **(**Sigma-Aldrich-Copper (II) oxide); CoCl_2_·6H_2_O (Sigma-Aldrich-Cobalt (II) chloride hexahydrate); Nitric acid (Sigma-Aldrich-ACS reagent, 70%); Formamide (Sigma-Aldrich-ACS reagent, ≥99.5%); DI water. All the chemical reagents were of analytical grade and used without any further purification.

**Preparation of Co-NC@Cu.** Synthesis of Co-NC@Cu involves several steps. Initially, 1g of CuO is dispersed in 30 mL of formamide (HCONH_2_) and stirred for 30 min until the solution is uniformly colored. Subsequently, 100 mg of CoCl_2_ (corresponding to 183.3 mg of CoCl_2_·6H_2_O) is added to the solution and stirred for 2 h. Then, the mixture of 30 mL solution is transferred into a 100 mL autoclave for hydrothermal treatment at 180°C for 12 hours. Under these reaction conditions, formamide converts into amorphous nitrogen-doped carbon (NC) material. After the hydrothermal reaction, the material is washed 4 times with 1:1 ethanol and water, with centrifugation at 7800 revolutions per minute (rpm). The washed material is then treated with 0.1 mM dilute nitric acid 3 times, followed by water rinsing to remove the nitric acid. Subsequently, the material is dried in a vacuum oven at 60°C for 12 hours. The dried powder sample is ground and placed into a quartz boat, followed by annealing in an Ar environment in a tube furnace. The reaction temperature is set at 400°C for 4 hours with a heating rate of 10°C per min. Finally, the resulting sample is ground again to complete the synthesis process.

**Preparation of NC@Cu.** The synthesis steps for NC@Cu are same as Co-NC@Cu, except for adding CoCl_2_.

**Sample characterization**

X-ray absorption spectroscopy (XAS) experiments were performed on the Medium Energy X-ray Absorption Spectroscopy (MEX-1) beamline stationed at the Australian Synchrotron (AS). The X-ray absorption near edge structure (XANES) and extended X-ray absorption fine structure (EXAFS) data for Co K-edge were acquired under ambient conditions. The subsequent interpretation of XAS data was facilitated using the ATHENA software suite.^[1]^ The surface analysis of X-ray photoelectron spectroscopy (XPS) was collected by an Al Kα X-ray source, 1486.6 eV (Kratos AXIS Ultra DLD). A FEI Verios scanning electron microscope (SEM) and equipped with Everhart-Thornley and immersion in-lens detectors for secondary electrons detection, was used to image the morphology of the samples. The same system was used to acquire cathodoluminescence signals by using a Gatan MonoCL4 Elite system. Full spectrum CL maps were acquired using a charge-coupled device (CCD). XRD patterns were acquired by Bruker X-ray diffractometer (Cu Ka). EPR spectra were acquired by a Bruker E500 spectrometer equipped with a Bruker ER 4122 SHQE resonator. Light source is 300 W Xenon lamp from PerfectLight. Gas products were analyzed by Nexis GC-2030 (Shimadzu Scientific instruments) which is equipped with a thermal conductivity detector (TCD), and a flame ionization detector (FID) with the ShinCarbon column. TRPL curves are captured at room temperature using an Horiba iHR 320 mm spectrophotometer equipped with a compact single-photon silicon detector (PPD-900, detection range between 350-920nm). A 485-nm picosecond pulse laser (Horiba DeltaDiode) is used as an excitation source. The laser light is focused on the samples through an Olympus BX53 microscope equipped with a 50x objective lens and an X-Y micropositioner. Micro-Raman spectroscopy measurements are conducted using a Horiba Labram system, which is equipped with confocal optics, a 532 nm diode-pumped solid-state (DPSS) laser, and a charge-coupled device (CCD) Si detector with detection range of 540-1000 nm. The laser light is focused onto the samples through a PL FLUOTAR microscope objective (50x magnification and a 0.55 numerical aperture). The excitation power directed onto the sample remains consistent across all measurements unless specified otherwise in the manuscript. The Co content in different samples was measured by an Agilent ICP-OES 5110. The powder samples were digested with 10% HNO_3_ to obtain the accurate metal compositions.

**The finite difference time domain (FDTD) simulations**

The theoretical finite difference time domain (FDTD) simulations were all performed by performing Lumerical (FDTD solutions, Lumerical Solutions, Inc., Vancouver, Canada) software. The optical parameters of Cu and Co-NC used in FDTD simulation were calculated from the results of DFT calculations. We assumed that the background medium was seawater with an index of refraction of 1.34. A total field-scattered field (TFSF) source of light consisting of plane waves was used as the incident light source. A uniform grid size of 1 nm (x, y, and z) was accepted to ensure the accuracy of E-field calculations. A frequency-domain field distribution monitor was used to evaluate the E-field distribution. The perfectly matched layer (PML) absorbing boundary conditions was considered. The E-field magnitude from the incident light was received by following steps ^[2]^:

*I*_ave_ = *cϵ_0_E_0_*^2^/2

*E*_0_ = (2*I*_ave_ /*cϵ_0_*)^1/2^

where I_ave_ is the intensity of incident solar simulator (600 mW/cm^2^), *c* is the speed of light, *ε*_0_ is the permittivity of free space, and *E*_0_ is the maximum E-field strength.

**Density functional theory (DFT) calculations**

The DFT calculations were studied by using the Vienna ab initio Simulation Package (VASP) code ^[3-4]^. Projector Augmented Wave (PAW) pseudopotentials ^[5]^ was used to deal with the core electrons and the Perdew-Burke-Ernzerhof (PBE) function of the generalized gradient approximation (GGA) ^[6]^ was used to describe electron exchange and related interactions. The cutoff energy of the plane wave basis was set to 420 eV. The convergence criteria for energy and force are set to less than 10^-4^ eV and 10^-2^ eV/Å, respectively. In addition, the van der Waals (vdW) correction was considered for the dispersion correction using Grimme's DFT-D3 method.^[7]^ For calculating optical properties of Cu and Co-NC, the Brillouin zone was sampled with 30 × 30 × 30 and 10 × 10 × 1 Monkhorst-Pack grids, respectively. The supercells are used to construct slab model for calculating free energy to avoid the interactions caused by periodicity. If mentioned, the E-field is included into the Gibbs energy calculations of reaction steps. This information could not be found anywhere The vacuum of 15 Å was set to avoid interlayer interactions. For calculating free energy, the Brillouin zone was sampled with a 3 × 3 × 1 Monkhorst-Pack grids. The reaction transition state (TS) was located using the Climbing Image Nudged Elastic Band method (Cl-NEB) between each state.^[8]^

**Photocatalytic experiments**

The photocatalytic CO_2_ reduction reaction was carried out in a liquid-solid phase reaction in a quartz reactor tightly sealed by a septum. The reaction was conducted under light irradiation of a 300 W Xe lamp (PLS-SXE300D, Perfect Light) with AM 1.5G filter without the use of any noble metal and sacrificial agents. Typically, 2 mg of the sample was dispersed in 10 ml artificial seawater and was sonicated (B2500R-DTH, Branson) for 10 min. The solution was then transferred into a reaction vessel and stirred. Subsequently, the reactor was sealed and purged with pure Ar for 15 minutes to remove air. Finally, the Xenon lamp was turned on to initiate the photocatalytic reaction. Following completion of the photocatalytic reaction, the reactor was allowed to cool, and GC measurements were conducted at room temperature.

For long-term stability testing, after each cycle of 23 hours of reaction, the light source was turned off. The reactor was then degassed again by purging with pure Ar for 15 minutes to remove gases from the previous reaction cycle, and the light source was reactivated to initiate the next reaction cycle.

**Solar-to-Hydrogen (STH) conversion efficiency**

To calculate Solar-to-Hydrogen (STH) conversion efficiency, we attempted to calculate STH considering the efficiency of the AM 1.5G solar spectrum. Based on previous work in solar-driven photocatalytic overall water splitting systems, the STH efficiency can be calculated as follows:^[9]^

$$\eta_{STH}=\frac{\text{n}_{\text{H}_{2}}\times\Delta A}{P_{solar}\times S\times t}\times100\%$$

where $\text{n}_{\text{H}_{2}}$ molar amount of the produced H_2_, ∆𝐴 is the Helmholtz free energy at the given temperature and volume, $P_{solar}$is the irradiance of 6 Sun (600 mW cm^-2^) of the AM 1.5G standard solar spectrum, S is the illuminating area (2 cm^2^), t is the reaction time.

**STEM-EDS & STEM-EELS methods and analysis**

Samples for aberration corrected scanning transmission electron microscopy (AC-STEM) were prepared by drop casting 5 µL of the sample dispersed in ethanol onto a Au TEM grid with lacey carbon support film.

AC-STEM imaging, energy-dispersive X-ray spectroscopy (EDS) and electron energy loss spectroscopy (EELS) were performed on a JEOL GrandARM300F2 operated at 300 kV and with a 24 mrad convergence angle. The collection semi-angles for the high angle annular dark field (HAADF) detector were 79-180 mrad and the bright field (BF) STEM detector had an outer collection semi-angle of 10 mrad. The instrument is equipped with dual 158 mm^2^ silicon drift X-ray detectors for EDS acquisition.

EELS mapping was collected using a Gatan Continuum (model 1069) spectrometer with a Gatan K3 direct electron camera. EELS mapping was performed with the high energy resolution entrance aperture which has a collection semi-angle of 72 mrad and spectra were recorded at a dispersion of 9 meV/channel. ADF images recorded alongside the EELS data were collected on the Gatan ADF detector (model 807) with collection semi-angles of approximately 50-150 mrad. The energy resolution is given by the FWHM of the zero-loss peak (ZLP) and was measured to be 0.405 eV.

For all spectra the zero loss and core loss regions of the spectrum were acquired simultaneously using the DualEELS mode and the ZLP was subsequently used to correct for any energy drift during the map due to scan or environmental instabilities during the acquisition.

The background zero loss was fitted and extracted using a non-linear least square fitting with a power law ($Ae^{-b\Delta E}$; where $A,b$ are fitting constants and $\Delta E$ is the energy loss). The maps shown are extracted from the map using 0.2 eV wide windows located on the observed plasmon peaks in the EELS spectrum centered at 2.0 eV, 2.3 eV and 2.6 eV.


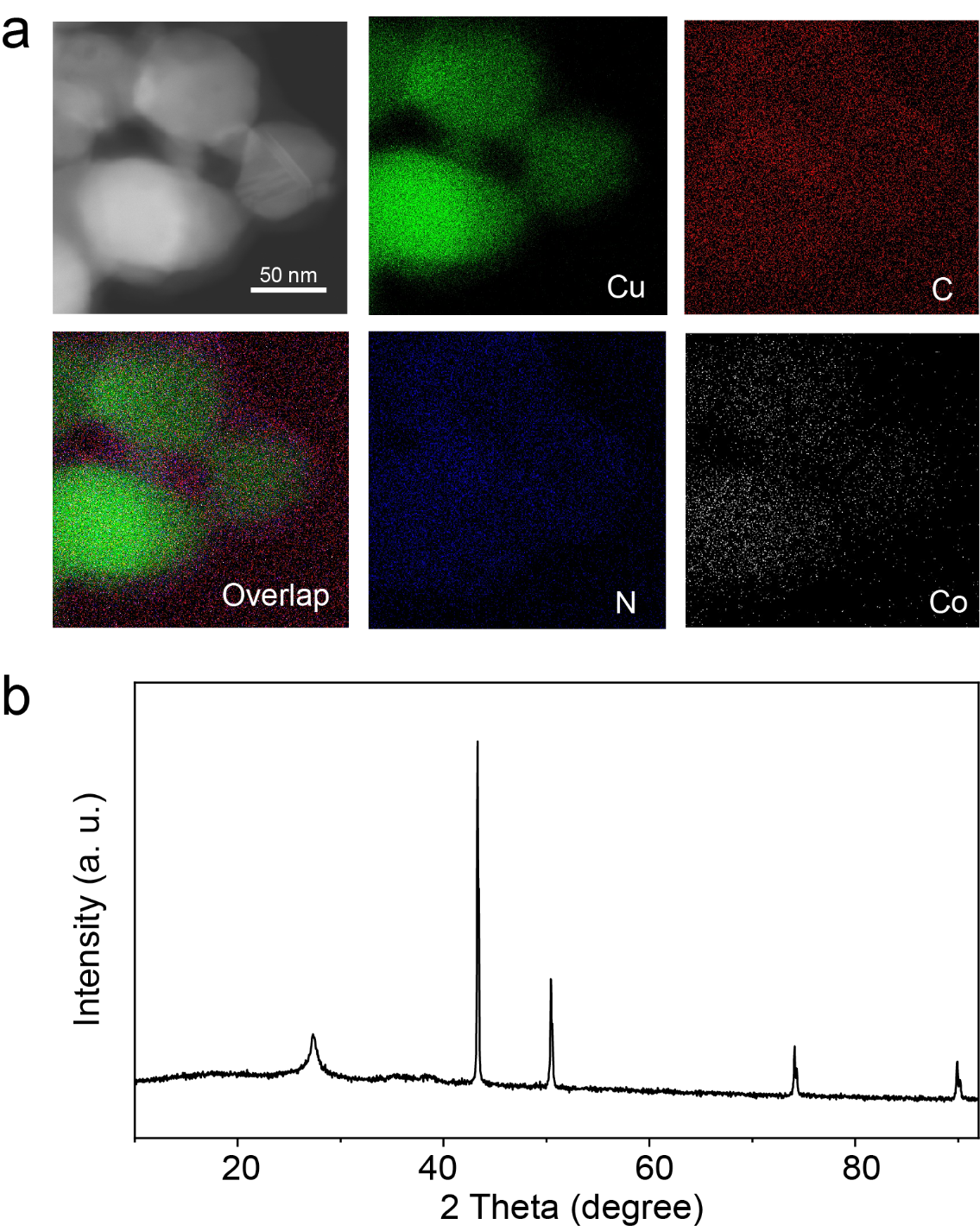


**Figure S1.** The HADDF-STEM and EDS element mapping of Co-NC@Cu for Cu, Co, N, and C. Due to the use of Ni/C mesh for sample preparation, the signal for C element mapping is affected. In the manuscript, we avoid the use of grids containing C for preparing TEM samples.


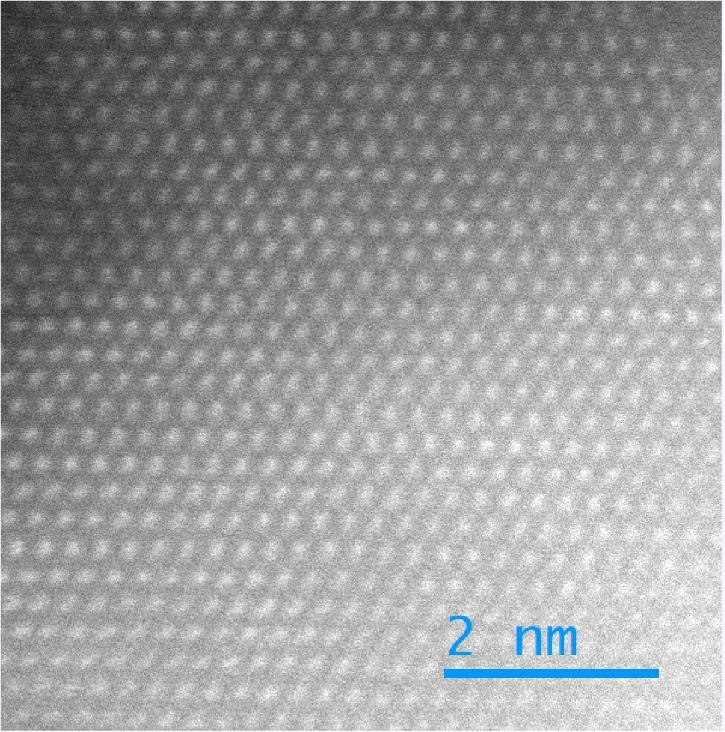


**Figure S2**. The AC HADDF-STEM local image of core Cu. The NP is viewed down a fcc<110> zone axis.


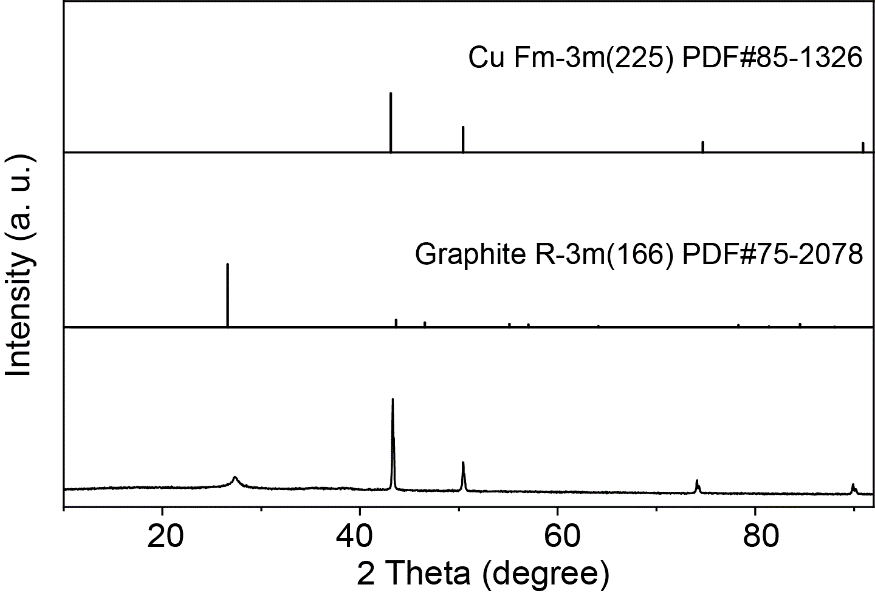


**Figure S3.** The XRD patterns of Co-NC@Cu after the 343-hour reaction.


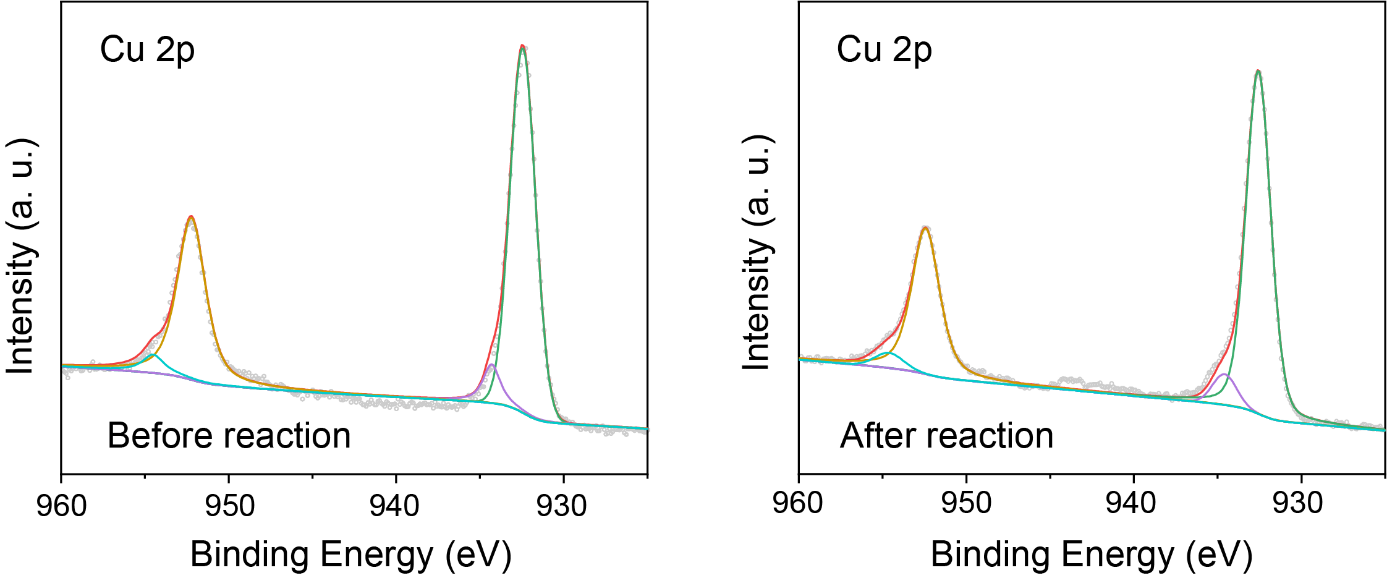


**Figure S4.** XPS spectra for Cu 2p before (left) and after (right) the reaction.


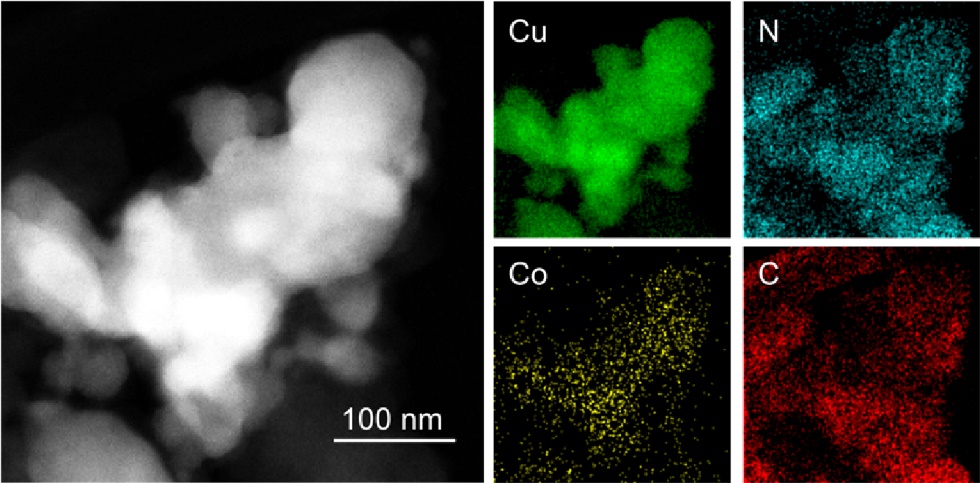


**Figure S5**. The HADDF-STEM and EDS element mapping of Co-NC@Cu for Cu, Co, N, and C after the reaction.


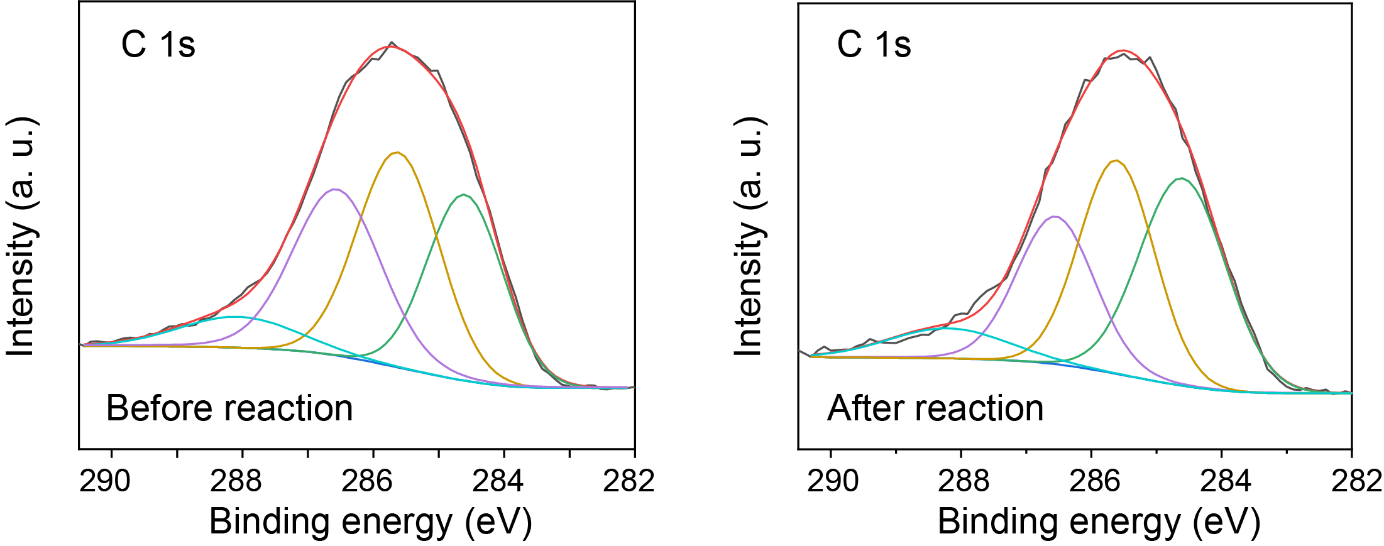


**Figure S6.** XPS spectra for C 1s before (left) and after (right) the reaction.


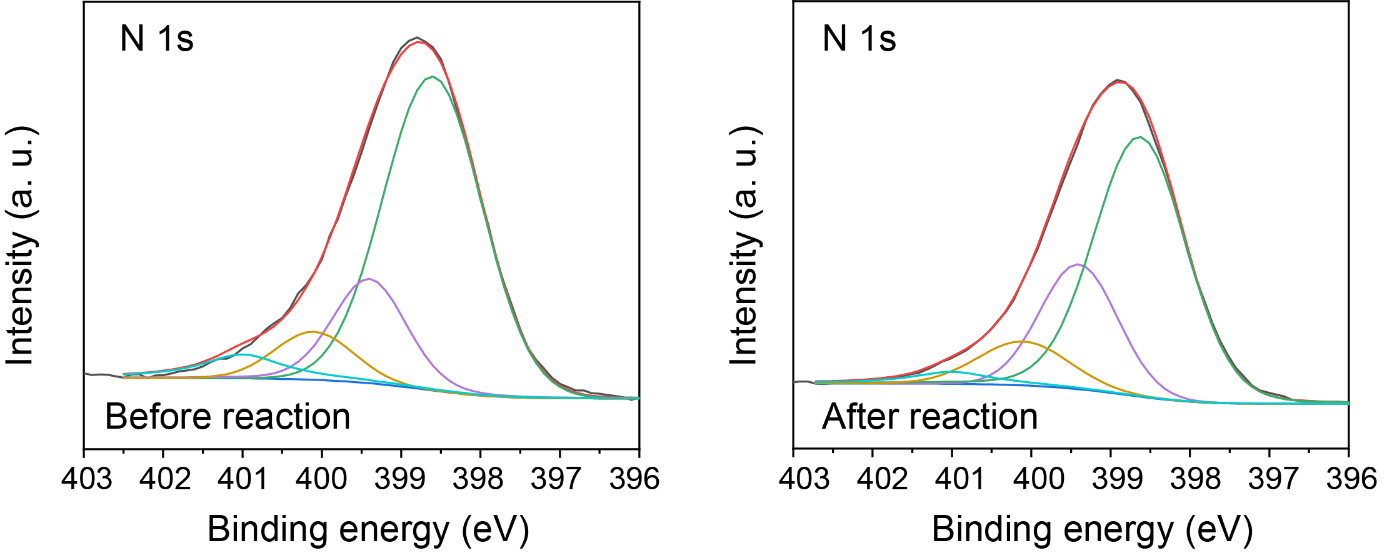


**Figure S7.** XPS spectra for N 1s before (left) and after (right) the reaction.


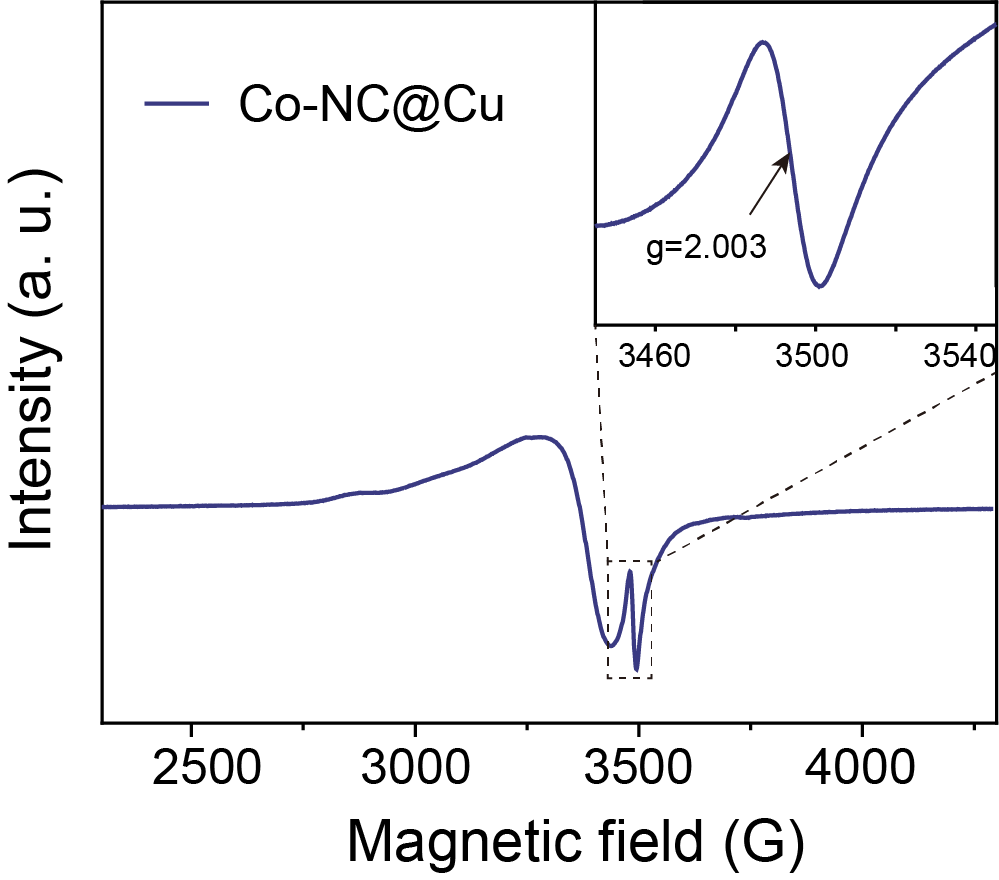


**Figure S8.** EPR spectra for Co-NC@Cu.


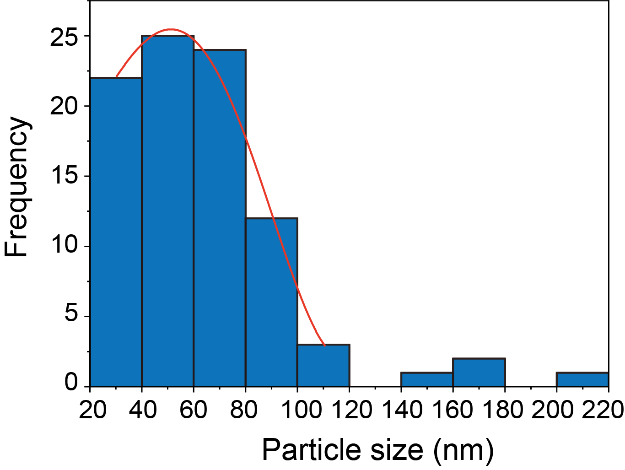


**Figure S9.** Size distribution histogram obtained from TEM images for Co-NC@Cu.


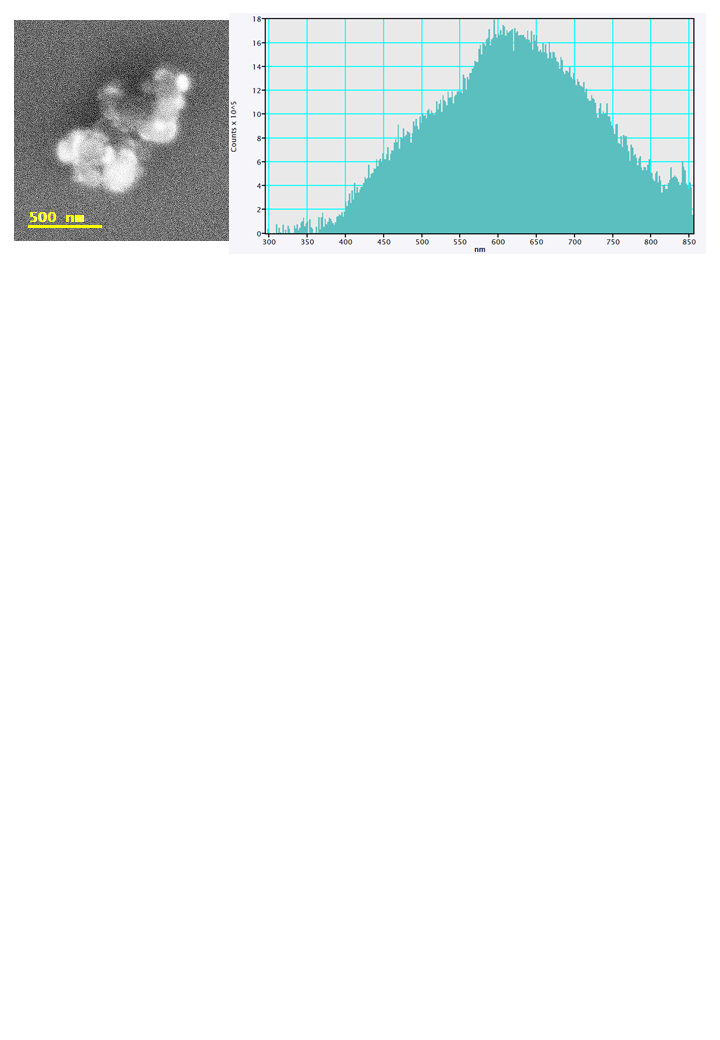


**Figure S10.** CL emission signal for Co-NC@Cu based on the corresponding SEM image and corrsponding emission spectra.


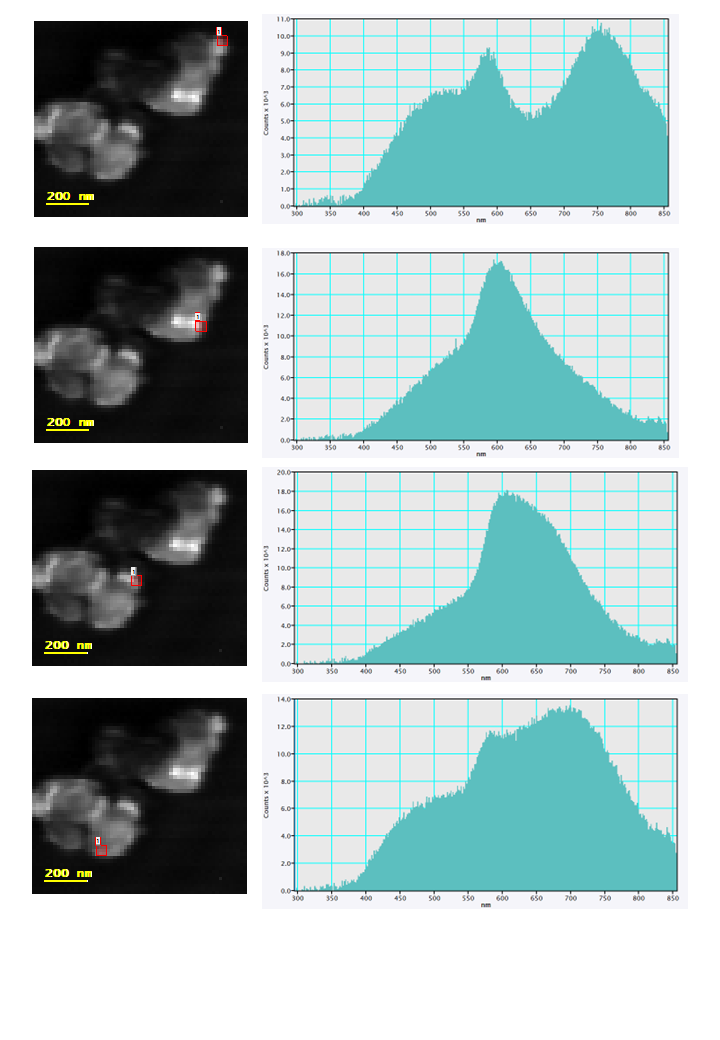


**Figure S11.** CL emission spectra at the different sites.


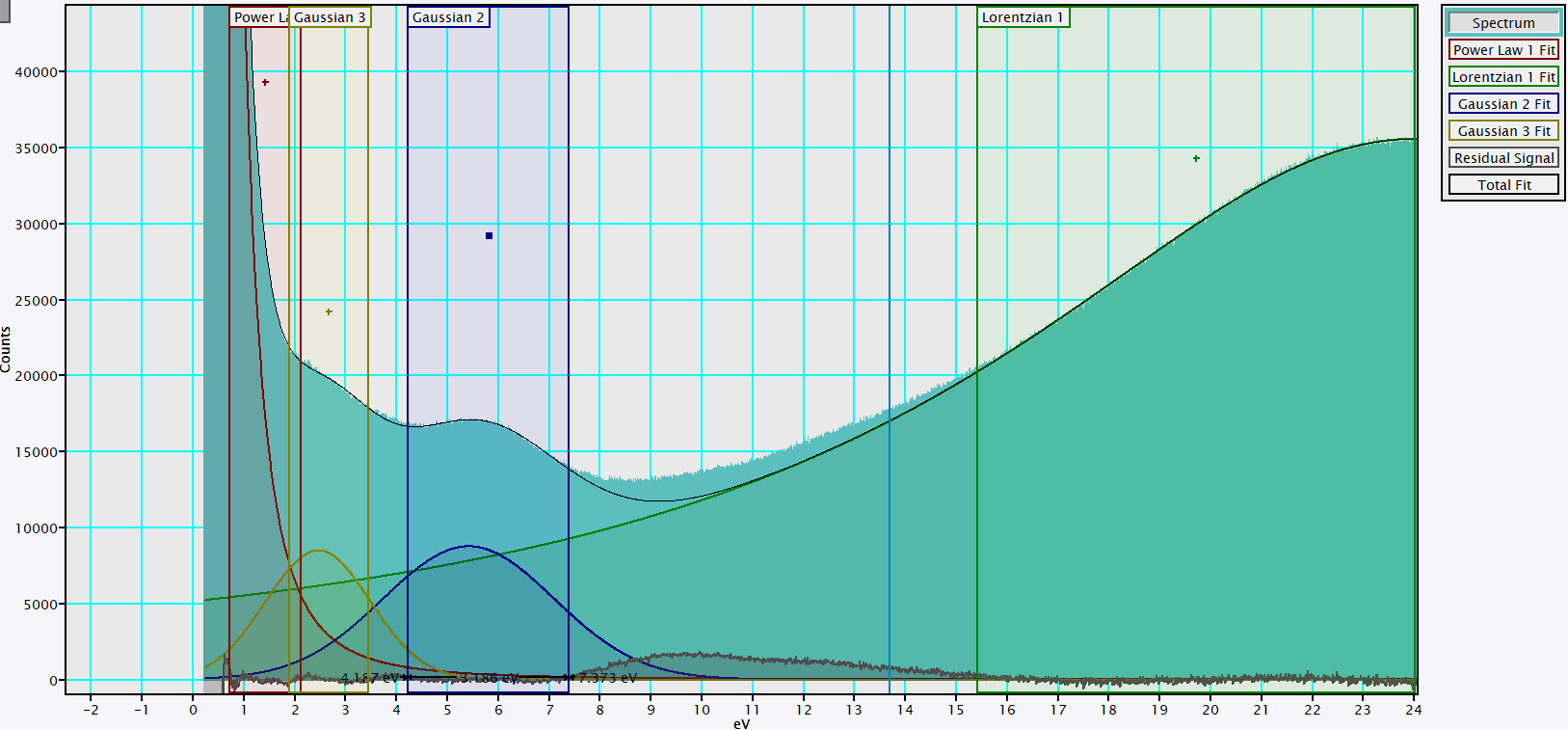


**Figure S12.** Fitting of line scan for EELS results. Fit the background as a power law spectrum; Fit the big plasmon at ~24 eV as a Lorentzian; One peak (orange curve) at ~2 eV fitted as gaussians; ~2 eV peak is attributed to the Cu NPs surface plasmon; Fitting shows good match, with small residuals.


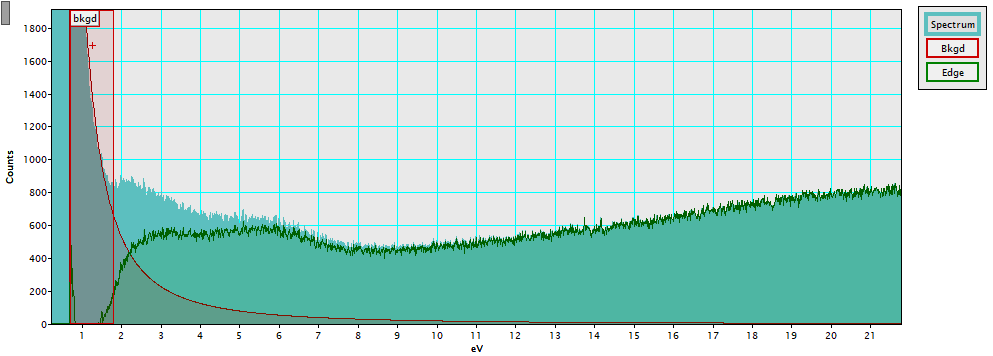


**Figure S13.** Plasmon signal mapping. Data was collected with a high dispersion for the highest resolution (0.4 eV). The ZLP was aligned post-acquisition, and the background was modelled as a power law leaving behind the signal from the plasmons. The broad peak is observed: at ~2 eV. The ~2 eV peak is attributed to the surface plasmon. The plasmon signals have been extracted and mapped using 0.2 eV wide windows located around the centre of the peak position as shown in the spectrum. Likewise, for the ~2 eV plasmon, however, as would be expected for a plasmon resonance this is more diffuse and extends further into the vacuum.


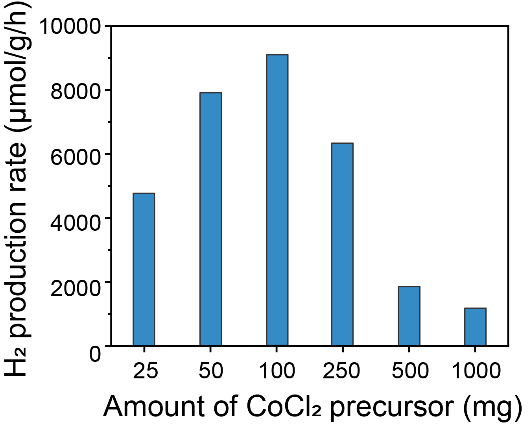


**Figure S14**. The reference experiments related to the optimization of Co loading.


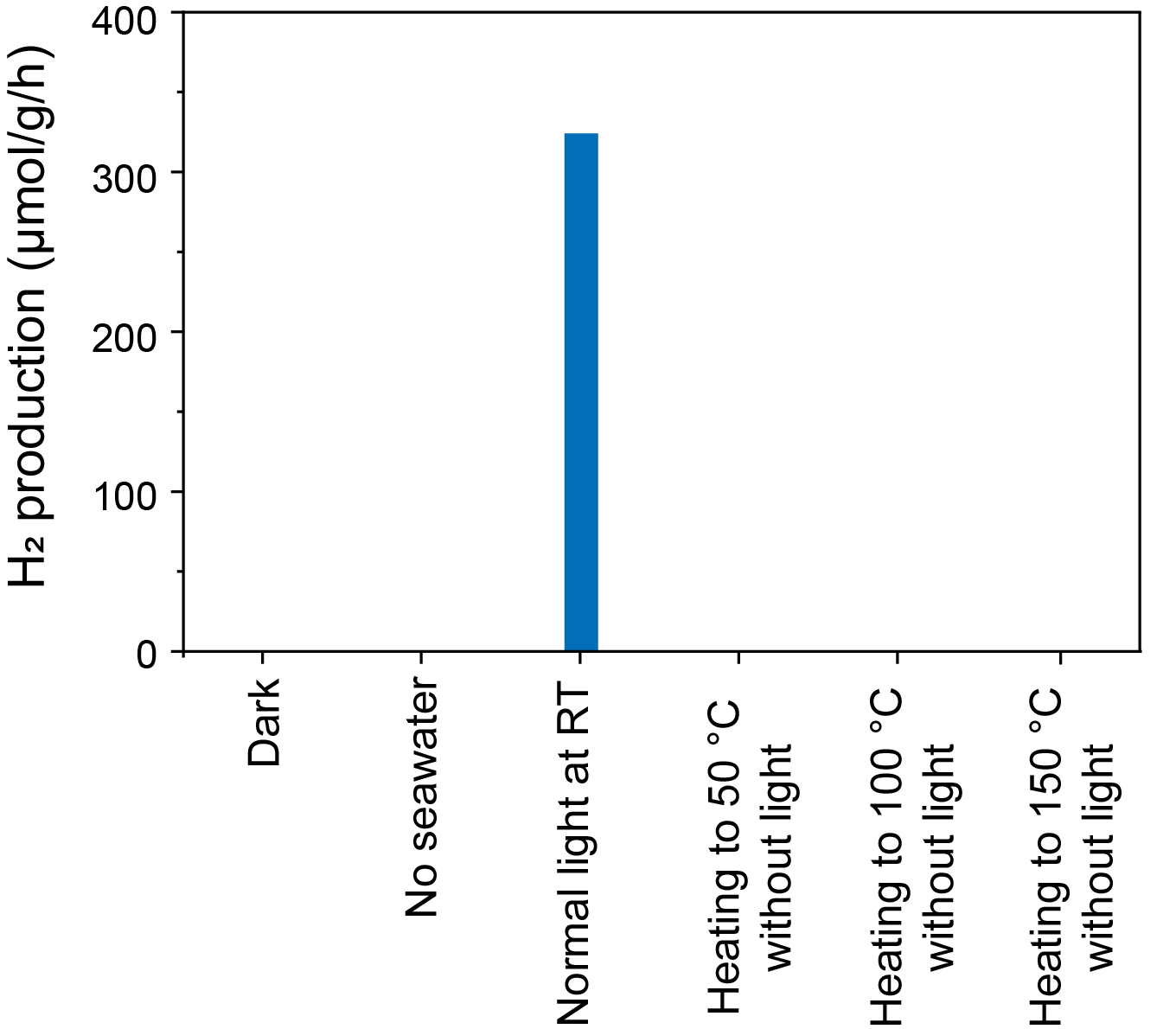


**Figure S15**. The reference for photocatalytic hydrogen production by seawater. Dark: without light and heat; No seawater: without any liquid, only powder samples; Normal light: 1 sun with 100 mW cm^-2^; RT: Room temperature; Heating was achieved by hot plate.


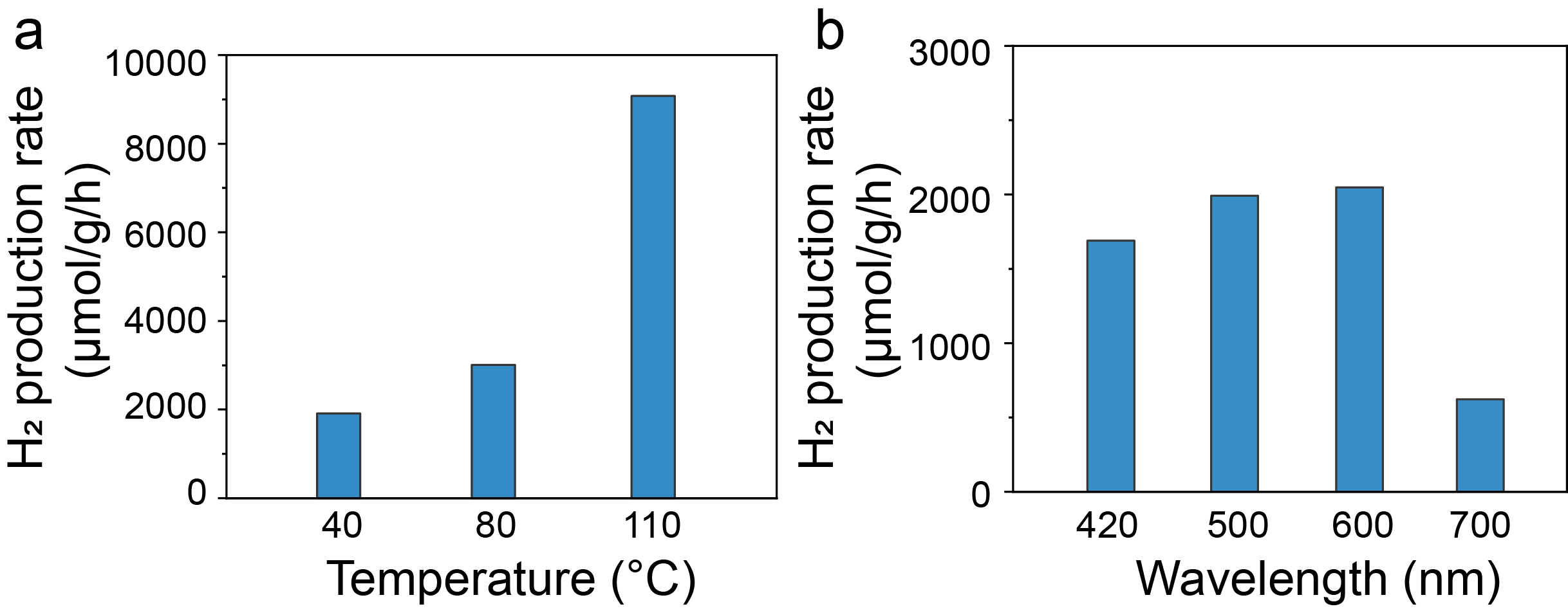


**Figure S16**. a) Temperature-dependent photocatalytic seawater splitting for H_2_ production. The experiment was studied by adjusting the reaction environment temperature using a water bath for the reactor while maintaining the same enhanced incident light conditions. b) Wavelength-dependent photocatalytic seawater splitting for H_2_ production


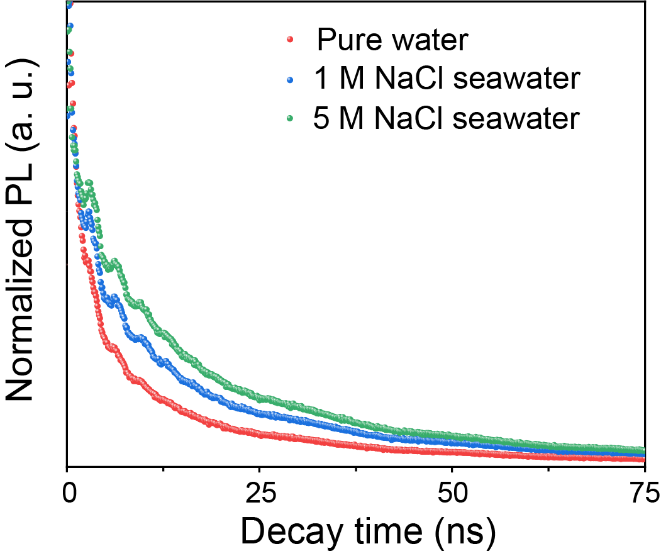


**Figure S17.**  TRPL spectra of the Co-NC@Cu tested in NaCl solutions at the concentrations of 0, 1, and 5 mol L^−1^, respectively. The excitation wavelength is 485 nm.


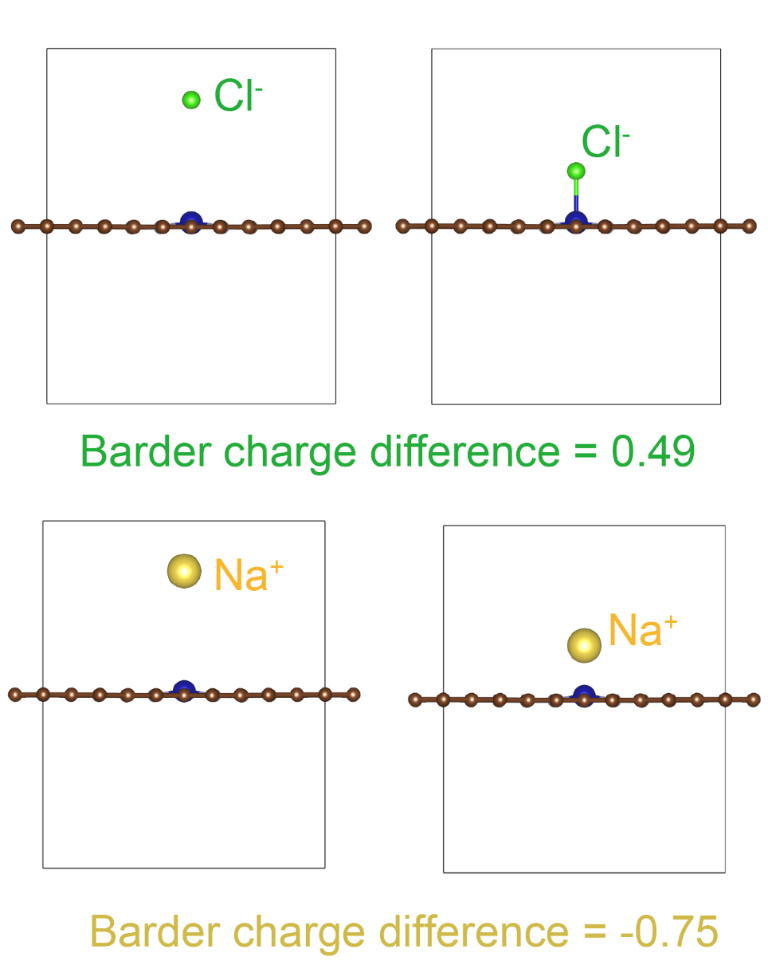


**Figure S18.** Calculated structures and Bader charges of Cl^-^ and Na^+^ adsorption on the Co-NC.

**
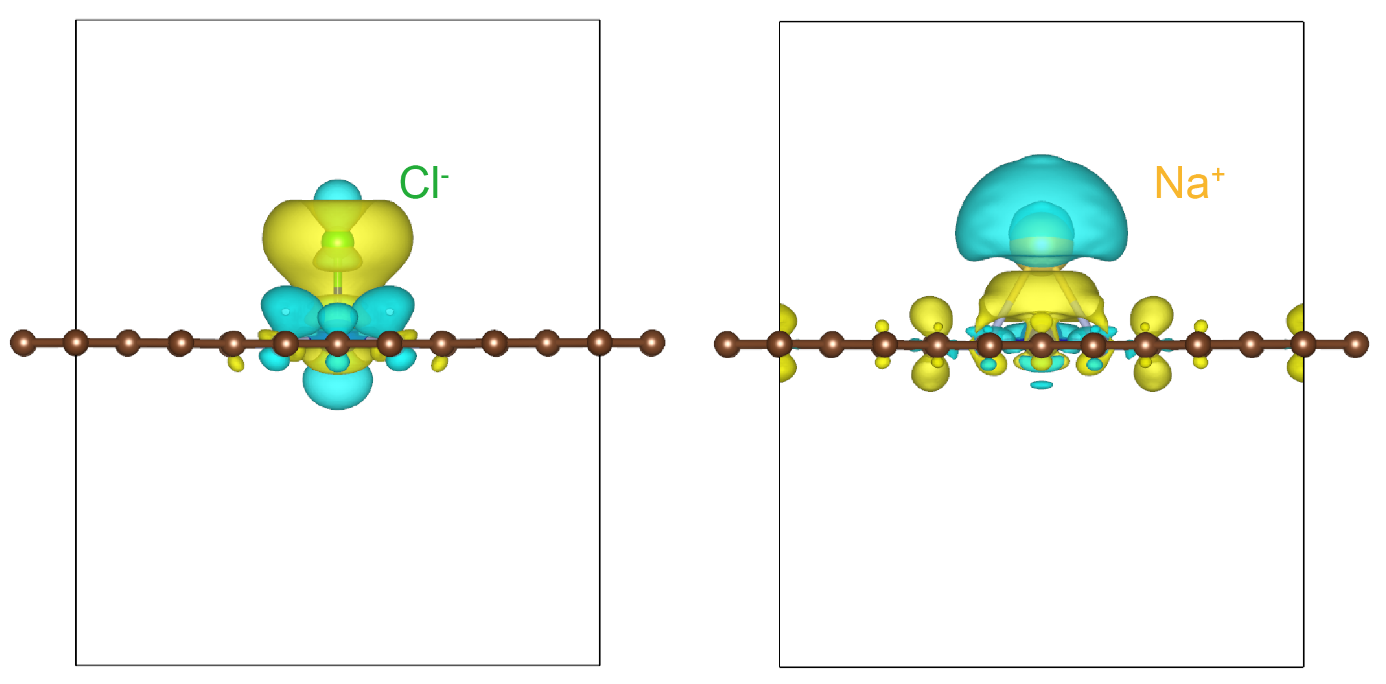
**

**Figure S19**. Calculated charge density difference plots for Cl^-^ and Na^+^ adsorption on the Co-NC. The charge density isosurfaces are plotted at a value of 0.001 e/Å^3^. Blue and yellow isosurfaces represent charge loss and charge accumulation, respectively.


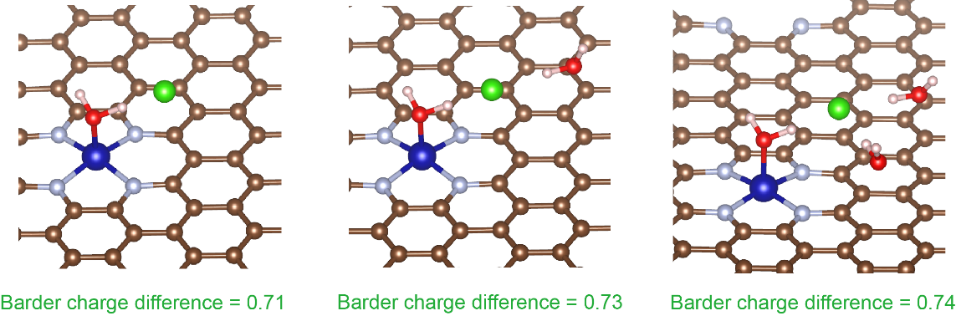


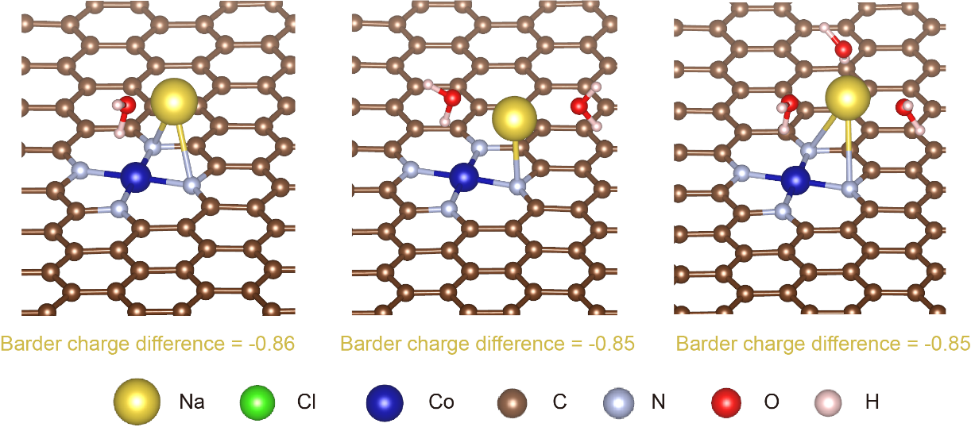


**Figure S20.** Calculated structures and Bader charges of hydrated Cl^-^ and Na^+^ adsorption on the Co-NC. The results show that hydrated Na^+^ ions are more inclined to adsorb onto the active sites around the Co-N-C coordination environment rather than the Co top site. Simultaneously, considering the adsorption of hydrated Cl^-^ ions, the competitive process between Cl^-^ and H_2_O for the Co top site using a slab adsorption model. The results indicate that H_2_O molecules preferentially occupy the Co top adsorption site.


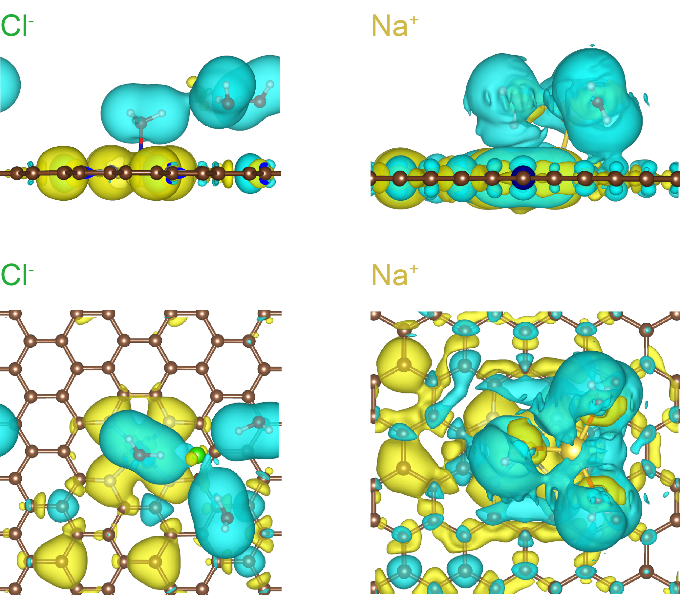


**Figure S21**. Calculated charge density difference plots for hydrated Cl^-^ and Na^+^ adsorption on the Co-NC. The charge density isosurfaces are plotted at a value of 0.01 e/Å^3^. Blue and yellow isosurfaces represent charge loss and charge accumulation, respectively.


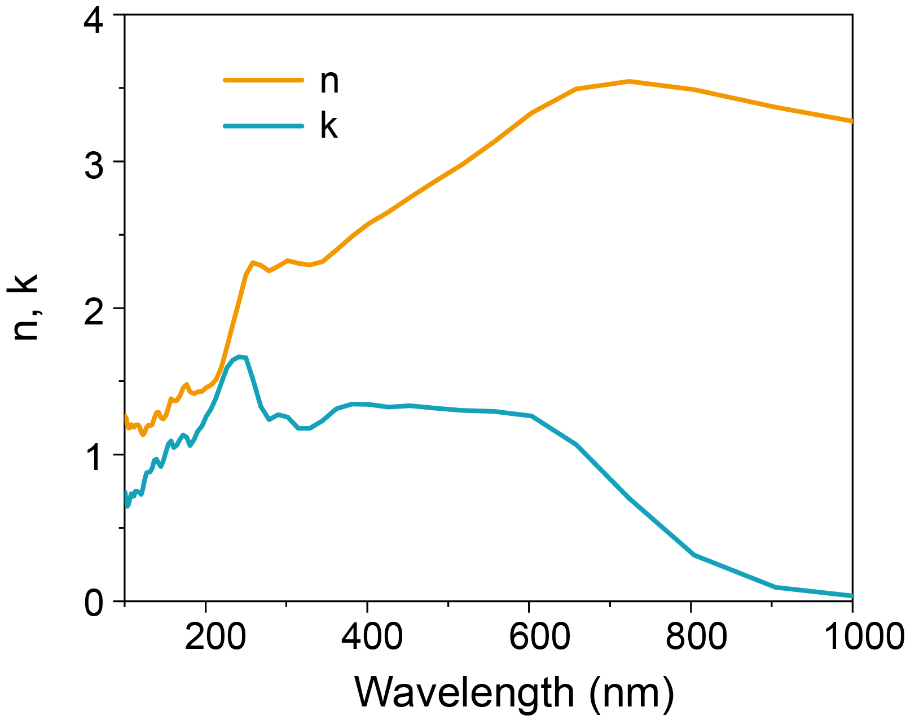


**Figure S22.** Refractive index (n) and extinction co-efficient (k) versus wavelength of Cu.


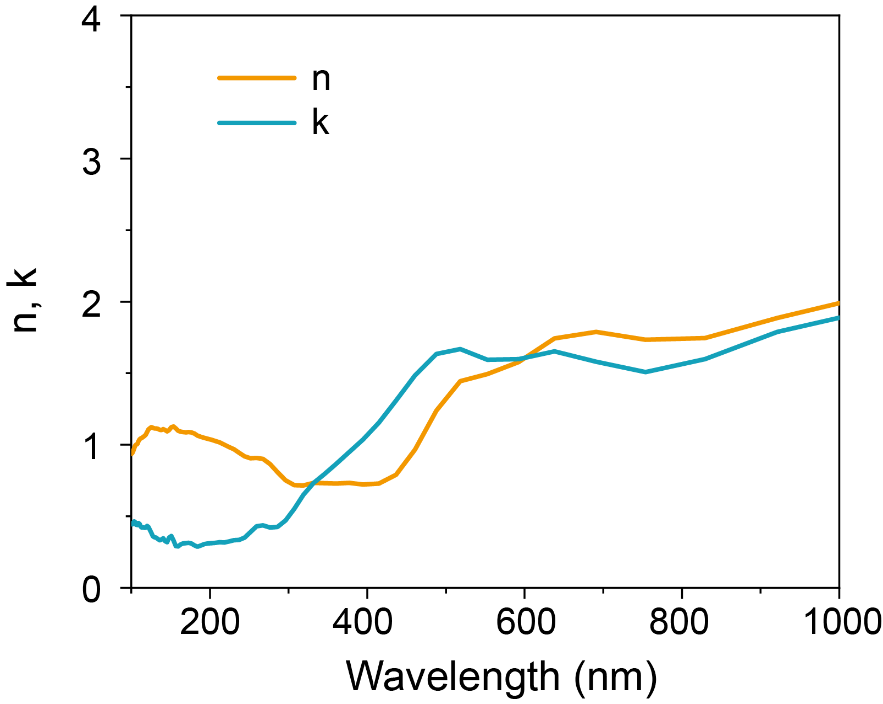


**Figure S23.** Refractive index (n) and extinction co-efficient (k) versus wavelength of Co-NC along *in-plane* direction.


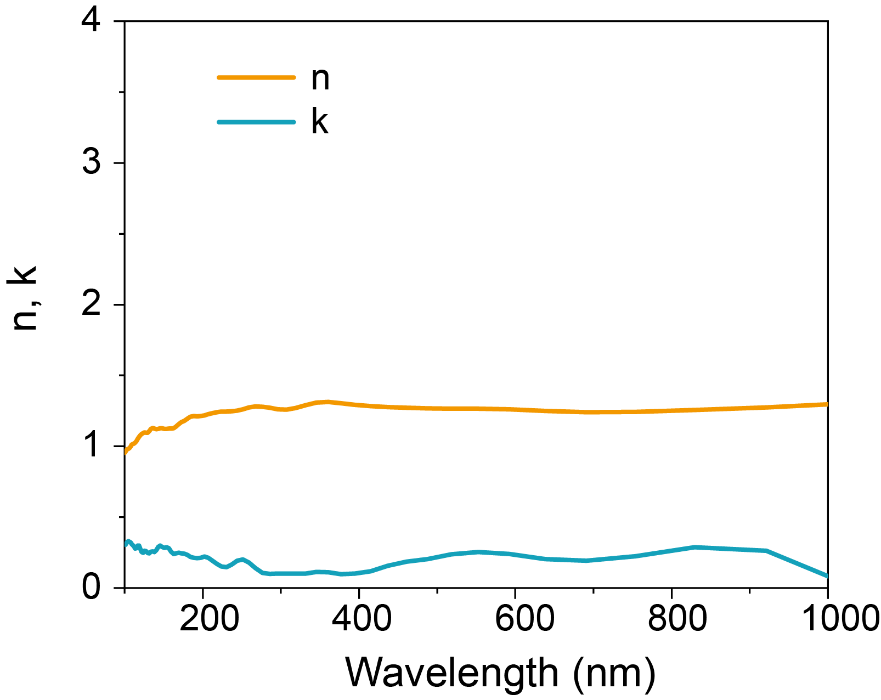


**Figure S24.** Refractive index (n) and extinction co-efficient (k) versus wavelength of Co-NC along *out-of-plane* direction.


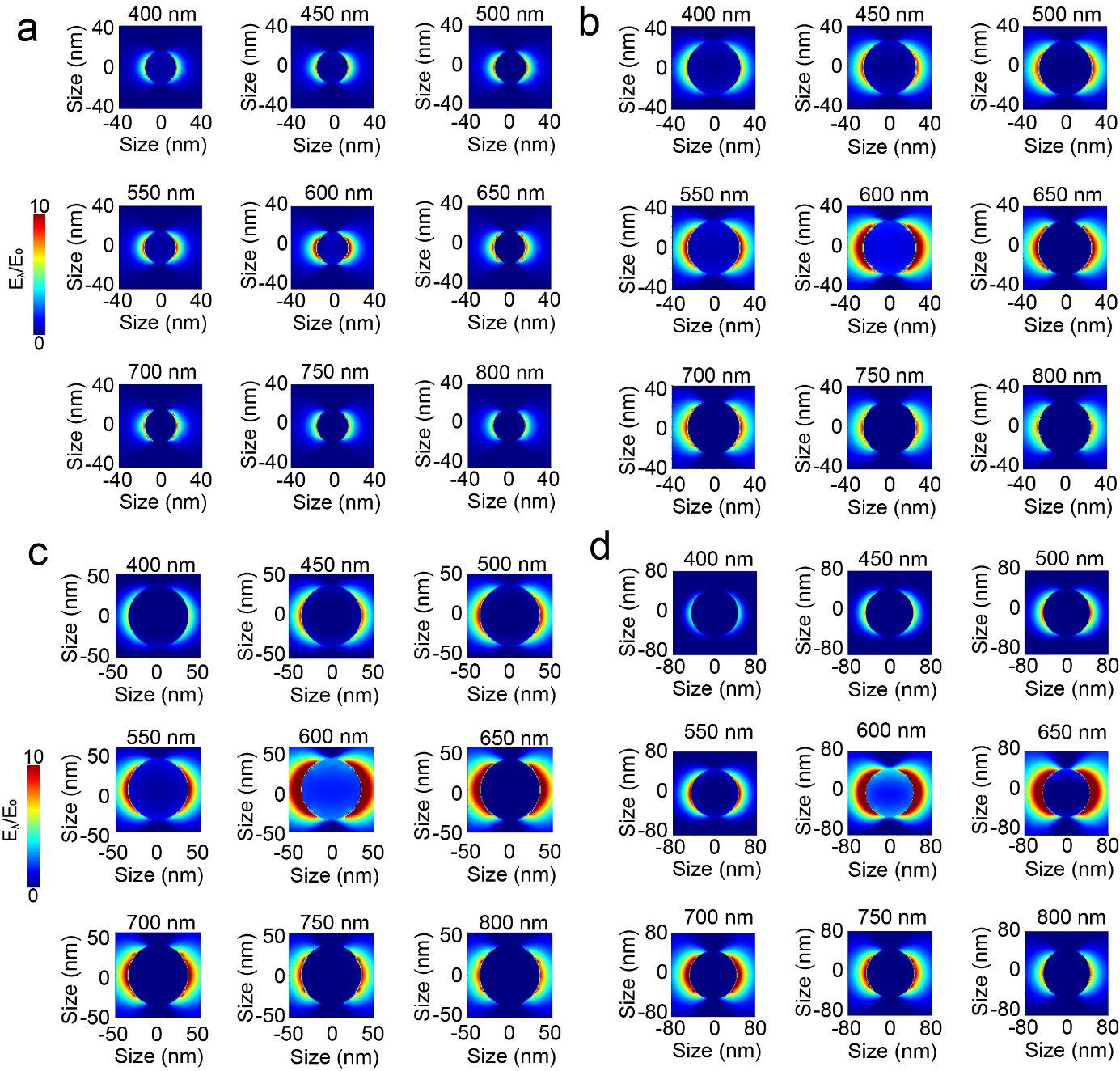


**Figure S25.** The simulated E-field distribution of Cu NPs under light irradiation at different wavelength ranges using FDTD simulations for a) 30 nm, b) 50 nm, c) 70 nm and d) 90 nm Cu NPs.


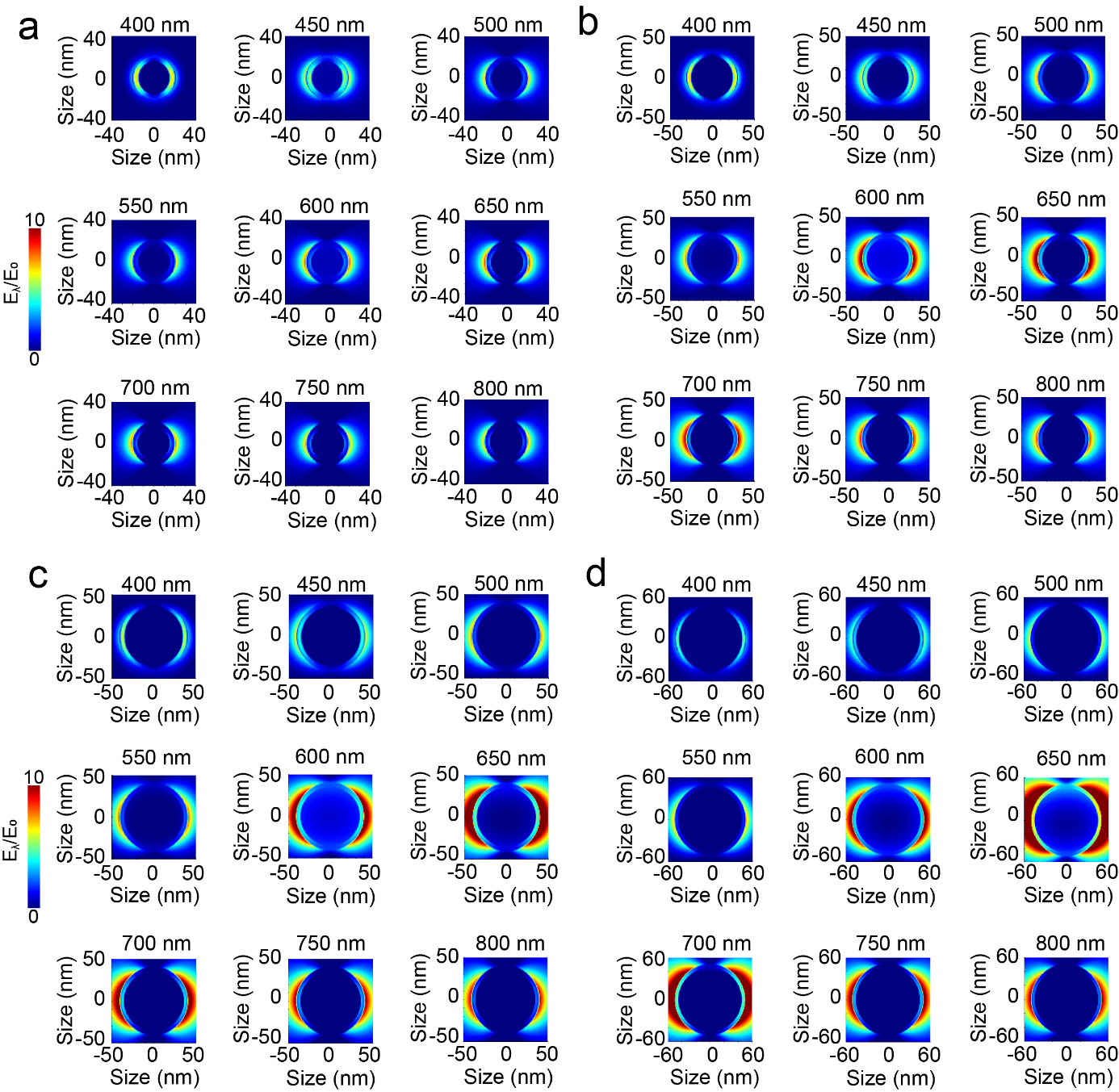


**Figure S26.** The simulated E-field distribution of Co-NC@Cu NPs with 5 nm thickness of NC layer under light irradiation at different wavelength ranges using FDTD simulations for a) 30 nm, b) 50 nm, c) 70 nm and d) 90 nm Cu NPs.


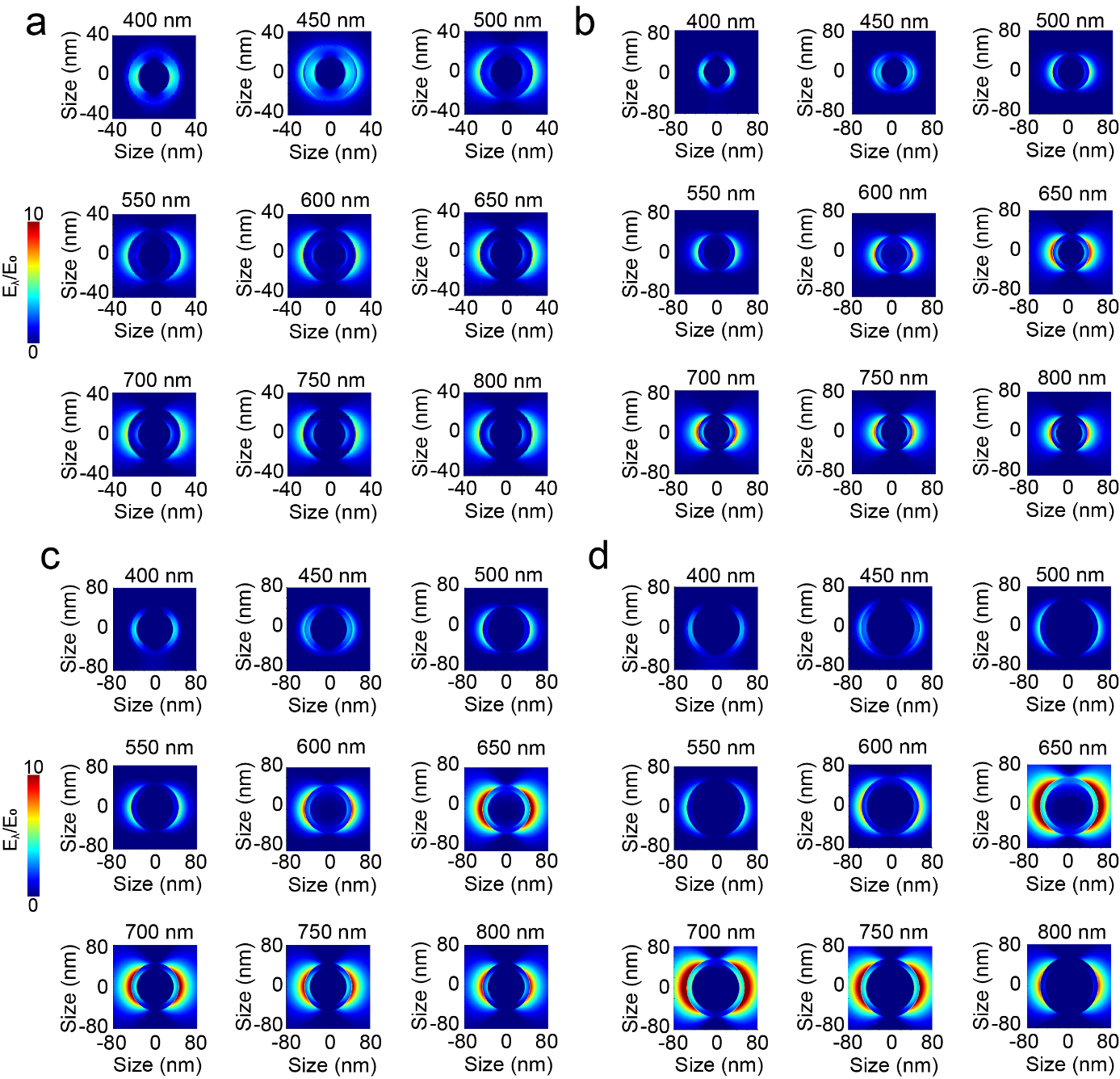


**Figure S27.** The simulated E-field distribution of Co-NC@Cu NPs with 10 nm thickness of NC layer under light irradiation at different wavelength ranges using FDTD simulations for a) 30 nm, b) 50 nm, c) 70 nm and d) 90 nm Cu NPs.


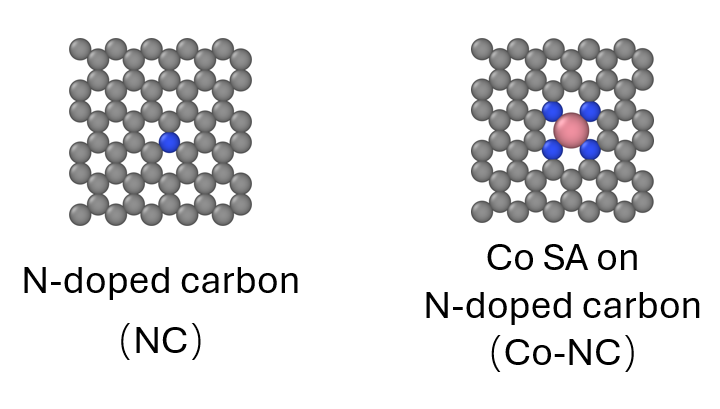


**Figure S28.** DFT modeling for NC and Co-NC. The colors of elements represent: Co, pink; N, blue; C, gray


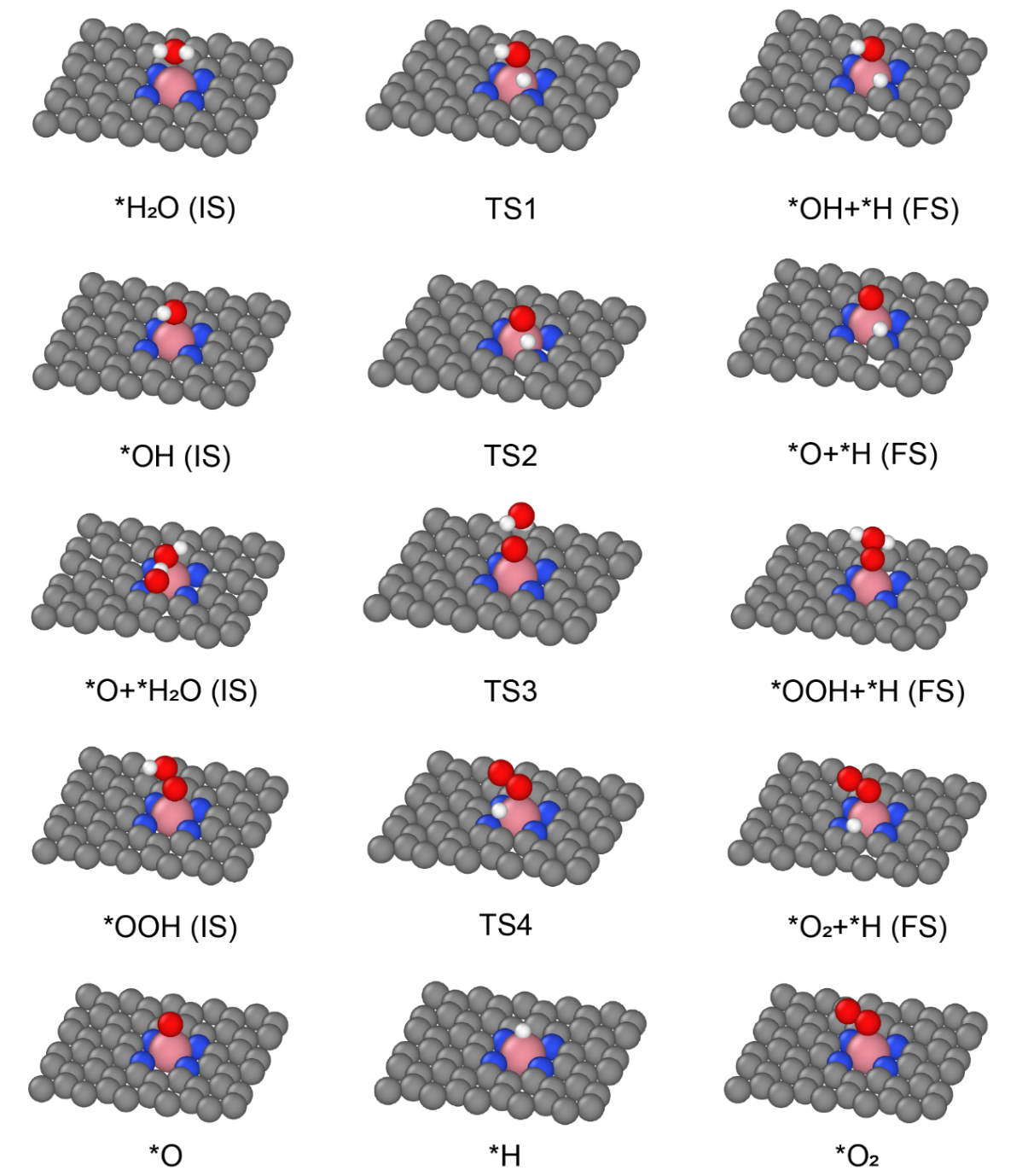


**Figure S29.** The optimized structure with intermediates adsorption for Co-NC@Cu. The colors of elements represent: Co, pink; N, blue; H, white; O, red; C, gray.

**Table S1.** Summary of photocatalytic hydrogen production using artificial and natural seawater without sacrificial agents (biomass-based catalysts were not included).

| Catalysts | Co-catalyst | Production rate  μmol/g/h | Reaction time  of stability test | Reference citation |
| --- | --- | --- | --- | --- |
| Co-NC@Cu | / | 9080 | 343 h | **This work** |
| Porous brookite TiO_2_ nanoflutes | Pt | 7200 | over 120 h | ^[10]^ |
| super-hydrophilic TiO_2_ | / | 6590 | 100 h | ^[11]^ |
| p-type (In)GaN | Rh/Cr_2_O_3_ | 6150 | / | ^[12]^ |
| TiO_2_ | Pt | 1476 | / | ^[13]^ |
| Ru-In SA/TiO_2_ | / | 1348 | 12 h | ^[14]^ |
| ZCS_0.1_ | / | 1145 | / | ^[15]^ |
| La_2_Ti_2_O_7_ | NiO, Ni | 696 | / | ^[16]^ |
| MgTiP | / | 630 | 39 h | ^[17]^ |
| Cdots/g-C_3_N_4_ | / | 539 | 96 h | ^[18]^ |
| Rh/Cr_2_O_3_/(Ga_1-x_Zn_x_)(N_1-x_O_x_) | Rh | 333 | / | ^[19]^ |
| Fe_2_O_3_/C-TiO_2_ | / | 170.8 | 24 h | ^[20]^ |
| CoP–pZCS | / | 154.6 | 5 h | ^[21]^ |
| ZIS-P | / | 51.3 | / | ^[22]^ |
| Pt/GaP-TiO_2_-SiO_2_:Rh | Pt, Rh | 23.6 | 16 h | ^[23]^ |
| Pt/GaP-C_3_N_4_ | Pt | 16.4 | 16 h | ^[24]^ |
| CuO/nano TiO_2_ | / | 3.2 | / | ^[25]^ |

**Table S2**. The Co loadings of Co-NC@Cu, determined by inductively coupled plasma optical emission spectrometry (ICP-OES) analysis.

| Sample | Co  (wt %) |
| --- | --- |
| Co-NC@Cu-50 | 0.30 |
| Co-NC@Cu-100 | 0.40 |
| Co-NC@Cu-250 | 0.49 |

Note: As Co-NC@Cu-100 for example, -100 means the precursor amount is 100 mg used for synthesis.

**Table S3**. The fitting of TRPL about carrier lifetime. The average carrier lifetime was calculated by using the following equation:

$$\tau_{\text{average}}=\frac{(A_{1}\times\tau_{1}^{2}+A_{2}\times\tau_{2}^{2})}{(A_{1}\times\tau_{1}+A_{2}\times\tau_{2})}$$

|  | $\tau_{1}$ | *A_1_* | $\tau_{2}$ | *A_2_* | $\tau_{\text{average}}$ |
| --- | --- | --- | --- | --- | --- |
| Pure water | 1.67 | 1.02 | 15.62 | 0.31 | 11.99 |
| 1 M NaCl | 2.21 | 1.10 | 18.81 | 0.43 | 14.97 |
| 5 M NaCl | 5.52 | 0.47 | 25.2 | 0.38 | 21.00 |

The unit of $\tau$is ns.

**Table S4.** The relevant DFT calculation results of adsorption energies on various possible absorbers on NC and Co-NC.

|  | N-doped Carbon | | Co-N-doped Carbon | |
| --- | --- | --- | --- | --- |
|  | E_ads_ (eV) | G_ads_ (eV) | E_ads_ (eV) | G_ads_ (eV) |
| Slab | -551.94 |  | -537.42 |  |
| *O_2_ | -561.99 | -562.12 | -548.17 | -548.17 |
| *OOH | -565.88 | -565.55 | -552.11 | -551.80 |
| *O | -556.65 | -556.60 | -542.40 | -542.39 |
| *OH | -561.68 | -561.37 | -547.05 | -546.82 |
| *H_2_O | -566.24 | -565.84 | -551.86 | -551.38 |
| *H | -554.89 | -554.58 | -540.92 | -540.73 |

**Author Contributions**

Z. Sun carried out the sample synthesis, performance test, FDTD and DFT calculation, and wrote the draft. S. Cheng. carried out the review, drew the figures and co-wrote the draft. X. Jing carried out the SEM-CL measurement. K. Liu carried out XRD and Raman. Y.-L. Chen carried out the analysis of XPS. A. A. Wibowo and D. MacDonald carried out the TRPL and Raman. H. Yin carried out the GC measurement. M. Usman carried out the ICP-OES analysis. S. Cheong, R. F. Webster, R. D. Tilley carried out the HRTEM, STEM, EELS and reviewd the draft. L. Gloag reviewed and revised the draft. N. Cox carried out the EPR. Z. Yin conceived and supervised the project, analyzed data, and wrote manuscript. All authors contributed to the overall scientific interpretation and edited the manuscript.

**References**

[1] B. Ravel, M. Newville, *Journal of Synchrotron Radiation* **2005**, *12*, 537.

[2] E. Hecht, Wesley Publishing Company, 1987.

[3] G. Kresse, J. Furthmüller, *Computational Materials Science* **1996**, *6*, 15.

[4] G. Kresse, J. Furthmüller, *Physical Review B* **1996**, *54*, 11169.

[5] P. E. Blöchl, *Physical Review B* **1994**, *50*, 17953.

[6] J. P. Perdew, K. Burke, M. Ernzerhof, *Physical Review Letters* **1996**, *77*, 3865.

[7] S. Grimme, J. Antony, S. Ehrlich, H. Krieg, *The Journal of Chemical Physics* **2010**, *132*, 154104.

[8] G. Henkelman, B. P. Uberuaga, H. Jónsson, *The Journal of Chemical Physics* **2000**, *113*, 9901.

[9] Y. Li, H. Zhou, S. Cai, D. Prabhakaran, W. Niu, A. Large, G. Held, R. A. Taylor, X.-P. Wu, S. C. E. Tsang, *Nature Catalysis* **2024**, *7*, 77.

[10] S. Cao, T.-S. Chan, Y.-R. Lu, X. Shi, B. Fu, Z. Wu, H. Li, K. Liu, S. Alzuabi, P. Cheng, M. Liu, T. Li, X. Chen, L. Piao, *Nano Energy* **2020**, *67*, 104287.

[11] Z. Cheng, X. Zhang, C. Bo, Y. Sun, C. Li, L. Piao, *International Journal of Hydrogen Energy* **2024**, *55*, 542.

[12] X. Guan, F. A. Chowdhury, N. Pant, L. Guo, L. Vayssieres, Z. Mi, *The Journal of Physical Chemistry C* **2018**, *122*, 13797.

[13] J. Zhang, Y. Lei, S. Cao, W. Hu, L. Piao, X. Chen, *Nano Research* **2022**, *15*, 2013.

[14] H. Peng, T. Yang, H. Lin, Y. Xu, Z. Wang, Q. Zhang, S. Liu, H. Geng, L. Gu, C. Wang, X. Fan, W. Chen, X. Huang, *Advanced Energy Materials* **2022**, *12*, 2201688.

[15] W. Cao, Z. He, M. Dai, G. Wang, G. Huang, S. Wang, *ACS Applied Energy Materials* **2023**, *6*, 4715.

[16] S. M. Ji, H. Jun, J. S. Jang, H. C. Son, P. H. Borse, J. S. Lee, *Journal of Photochemistry and Photobiology A: Chemistry* **2007**, *189*, 141.

[17] C.-T. Lee, L.-I. Hung, Y.-C. Shih, J. C.-S. Wu, S.-L. Wang, C.-W. Huang, V.-H. Nguyen, *Journal of Environmental Chemical Engineering* **2021**, *9*, 104826.

[18] J. Liu, Y. Liu, N. Liu, Y. Han, X. Zhang, H. Huang, Y. Lifshitz, S.-T. Lee, J. Zhong, Z. Kang, *Science* **2015**, *347*, 970.

[19] K. Maeda, H. Masuda, K. Domen, *Catalysis Today* **2009**, *147*, 173.

[20] Y. Yang, L. Liu, Q. Qi, F. Chen, M. Qiu, F. Gao, J. Chen, *Catalysis Communications* **2020**, *143*, 106047.

[21] Y.-H. Chew, B.-J. Ng, J.-Y. Tang, L.-L. Tan, S.-P. Chai, *Solar RRL* **2021**, *5*, 2100016.

[22] B.-J. Ng, W.-K. Chong, L. K. Putri, X. Y. Kong, J. Low, H. W. Lee, L.-L. Tan, W. S. Chang, S.-P. Chai, *Journal of Materials Chemistry A* **2023**, *11*, 17079.

[23] H. V. Dang, Y. H. Wang, J. C. S. Wu, *Applied Catalysis B: Environmental* **2021**, *296*, 120339.

[24] H. V. Dang, Y. H. Wang, J. C. S. Wu, *Applied Surface Science* **2022**, *572*, 151346.

[25] A. J. Simamora, T. L. Hsiung, F. C. Chang, T. C. Yang, C. Y. Liao, H. P. Wang, *International Journal of Hydrogen Energy* **2012**, *37*, 13855.
